# Force-induced transition state rupture enables mechanistic control in aziridine mechanochemistry

**DOI:** 10.1039/d5sc04954g

**Published:** 2025-08-16

**Authors:** Anne Germann, Jan Meisner

**Affiliations:** a Institute of Physical Chemistry, Heinrich Heine University Düsseldorf 40225 Germany meisner@hhu.de

## Abstract

Besides long established thermal and photochemical activation of chemical reactivity, mechanical forces emerged as a further tool to drive reactions. Molecular motifs which undergo particular transformations under external force, so called mechanophores, are oftentimes small cyclic structures which can easily be activated due to their inherent ring strain. In the ring-opening of *cis*-substituted 4 π-electron mechanophores, the pulling force activates the Woodward–Hoffmann-forbidden disrotatory reaction, which can compete with the allowed conrotatory reaction. We introduce the concept of transition state rupture, a force-induced catastrophe which results in changing the preferred reaction pathway on the force-modified potential energy surface, controlling selectivity. By computing force-modified stationary points and reaction pathways for various linker-mechanophore combinations we rigorously investigate how the magnitude of the external force determines the mechanochemical mechanism. Using the concept of transition state rupture, we explain previous observations made in sonication experiments studying the activation of aziridine mechanophores, elucidating the reaction mechanisms and product selectivity.

## Abstract 2

Force-transferring substituents can excert control over mechanochemical ring-opening reactions, influencing selectivity between con- and disrotatory pathways. We investigate and explain selectivity through concepts derived from catastrophe theory.

## Graphical abstract

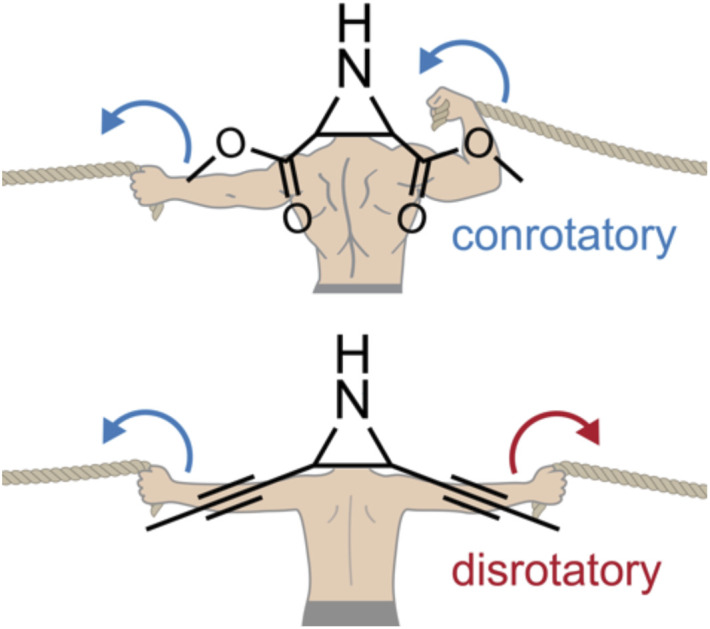

## Introduction

The activation of ring-opening reactions of strained cyclic molecules through the application of an external pulling force has been a key concept in the design of force-responsive structures for mechanochemists.^1–3^ An interesting feature of this mechanical activation is the ability of pulling forces to enable reactions considered symmetry-forbidden, which can take place if the atomic motions through which the reaction proceeds are favorable under force.^4–6^ The disrotatory motion, which is forbidden in systems with 4*n* π-electrons according to the rules of Woodward and Hoffmann (WH), can be systematically activated by applying a pulling force to a *cis*-substituted cyclic molecule.^7,8^ In this situation the WH-allowed conrotatory reaction competes with the mechanochemical disrotatory reaction.^9^ The selectivity is controlled both by electronic factors and by the magnitude of the mechanical force.^9^

The competition between thermal conrotatory and force-assisted disrotatory pathways has been probed both experimentally and computationally for various mechanophores, including cyclobutene (CBE), benzocyclobutene (BCB), and oxirane.^4,8,10–13^ Computational methods that simulate the effect of external pulling forces on molecules have been developed to support these ongoing investigations. Most prominent here are the force-modified potential energy surface (FM-PES) and external force is explicitly included (EFEI) methods, which directly modify the potential energy surface through an external force potential term.^8,14,15^ These methods allow the optimization of force-modified geometries and reaction paths, enabling the investigation of reaction mechanisms under mechanochemical conditions. This is a great improvement on the much simpler, yet still widely employed, constrained geometries simulate external force (CoGEF) method, which mimics a pulling force through geometry constraints.^16^ As the former methods include the external force as a separate potential term in the computations, distinctions between electronic and external forces can be made. This can provide significant insight into how a mechanophore's reactivity is modulated by external force.

**Fig. 1:**
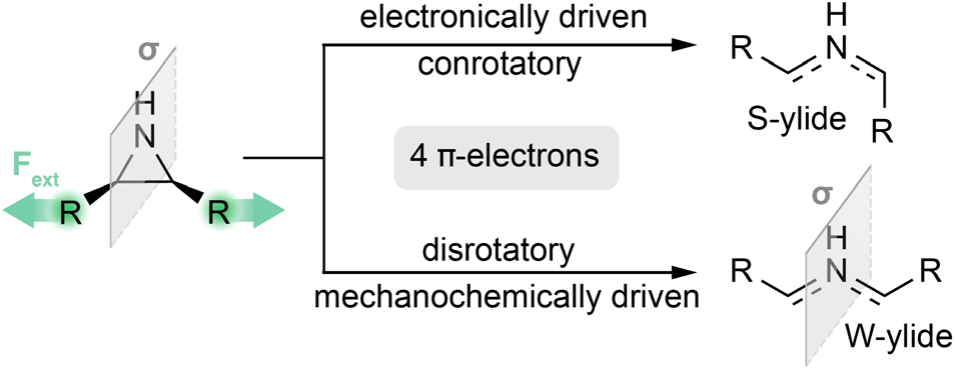
Electronically controlled and force-controlled ring-opening reactions on the example of a *cis*-substituted aziridine mechanophore. The mirror plane symmetry element present in the aziridine and W-ylide structure is indicated in grey.

One case where the mechanistic behavior under mechanochemical conditions needs further study is that of the aziridine mechanophore's ring-opening reaction. Under thermal conditions, this reaction produces an azomethine ylide with a zwitterionic structure. The product stereochemistry is torque-selective: the conrotatory reaction results in an S-shaped ylide structure, while the disrotatory reaction leads to a W-shaped product, see Fig. 1.^17–19^ As the stereochemistry of these ylides can affect follow-up reactions, it is important to understand the influence external force has on the reaction mechanisms. Aziridine-based mechanophores have been studied experimentally by Jung and Yoon, who have reported the force-induced ring-opening of *N*-methoxybenzene- and *N*-phthalimido-substituted aziridines.^20,21^ In both cases, the external pulling force was applied by sonicating solutions of polymers containing the mechanophore. Their first study, focusing on *N*-methoxybenzene aziridine, reported no isomerization from *cis* to *trans* or *vice versa* after mechanochemical activation of stereochemically pure polymers.^20^ When the same activation experiments were performed in the presence of dimethyl acetylenedicarboxylat (DMAD), a dipolarophile, cycloaddition products where obtained with high stereoselectivity. The reaction of *cis*-aziridine mechanophores with DMAD resulted in *trans*-configured adducts, while that of *trans*-aziridine yielded the *cis* product. This unexpected finding lead the authors to state that the reaction follows a ylide-free path. However, a conclusive mechanism that fully explains the observations has not been reported so far.^20^

In their second work on aziridine mechanochemistry, the same authors present the force-induced activation of *N*-phthalimido-aziridine.^21^ Here, a migration of the phthalimide group was observed, leading to a hydrolyzable imine product after sonication. From CoGEF calculations they suggest that an S-shaped ylide intermediate is formed as a result of the ring-opening reaction, which then undergoes the migration as a secondary reaction step.^16^ Still, they rightfully remain careful about drawing mechanistic conclusions from CoGEF results, and have instead opted to leave this question open for further studies. As the mechanochemical activation mechanism of this process was not described, it remains unknown whether the migration occurs in a single concerted step with the aziridine ring-opening or as a follow-up process to the ring-opening reaction.^21^ In the present paper, we will show that considering the competition between conrotatory and disrotatory pathways is the key to understanding the stereochemistry of these reactions.

Other mechanophores also pose questions of mechanistic selectivity under force. Multiple studies have shown that in CBE mechanophores, the conrotatory and disrotatory reaction paths can be competitive.^11,12,22^ Depending on the substitution pattern, the energetic gap between the WH-allowed conrotatory and the forbidden disrotatory transition state structure can be surprisingly small. As the activation energies of both of these ring-opening reactions are modified through the external force, understanding chemical selectivity can become a complex endeavor. Furthermore, studies on this subject hint at force-induced catastrophes, “tipping points” where a small change in external force causes a sudden drastic change in system behavior.^11,23^ Such topological catastrophes were investigated in detail for dihalo-cyclopropane mechanophores, where it was shown that the transition structures of conrotatory and dis-rotatory ring-opening reactions collapse into a single ambimodal transition structure when an external pulling force is applied.^24^

As early as 2003, computational studies have shown that the energetic gap between allowed and forbidden ring-opening mechanisms of CBE can be altered through the use of planarity constraints.^25^ Building on this, computations with planarity-enforcing geometric constraints have been able to predict a mechanistic switch from the WH-allowed conrotatory to the forbidden disrotatory reaction.^26^ In that study, a second order saddle point (SOSP) corresponding to the disrotatory ring-opening motion was found in the unrestrained molecule, which was then converted to a first order saddle point as greater planarity was enforced in the system. SOSPs are points on the potential energy surface that exhibit two distinct energy-lowering motions. First order saddle points, recognized as transition structures, allow only one energy-lowering motion for the system, which is the motion of the corresponding chemical reaction. The difference between second and first order saddle points is also evident in the eigenvalue spectrum of the Hessian matrix, where energy-lowering, *i.e.* concave-down, modes correspond to negative eigenvalues. In addition to this conversion of a SOSP to a transition structure, the conrotatory pathways were eliminated because the required out-of-plane motion became impossible for the system once the planarity constraint became too great. Here, the planarity constraint served as the catastrophe-inducing parameter in place of an external pulling force. All of these findings support the growing consensus that the clear-cut differentiation between “allowed”, *i.e.* energetically feasible, and “forbidden” high-energy mechanisms is much more diffuse than commonly assumed.^27,28^

In this paper, we will explore how an external force influences the competition between WH-favored and -disfavored mechanisms through the modification of stationary points. Furthermore, we will show how this behavior can be controlled through substituents, adding to the toolkit of mechanophore design. The paper is structured as follows: the first and second results section will present detailed mechanistic and topological investigations for the paradigmatic aziridine mechanophore, aiming to explain the mechanistic changes caused by external force. The third section then expands the study to other mechanophore cores and linker substituents in order to understand how the behavior can be systematically influenced. Finally, we will use the concepts established in the first three sections to explain the observations made in the experimental study of aziridine ring-opening reactions, where so far the mechanism has not yet been conclusively reported. Here, molecular dynamics studies were made to elucidate the post-transition state reactivity. All energies we report in this paper were computed with complete active space perturbation theory of second-order (CASPT2),^29^ using geometries optimized with density functional theory (DFT). The computational and methodological details and data can be found in the SI.

## Results and discussion

### The rupture force catastrophe of reactant structures

For simple mechanophores, reactivity under external force is mostly defined by a single dominant reaction. This is the case for *trans*-substituted 4π-electron mechanophores, where the atomic motions of the WH-favored conrotatory ring-opening reaction are aligned with the pulling force.^4^ One characteristic value often noted for mechanophores is the rupture force *F*_R_, which is the maximal external force before the reactant structure is no longer a stable minimum and the potential energy barrier of the force-activated reaction vanishes. This topology switch is a fold catastrophe, as the rupture force signifies a non-linear change in system behavior.^15^

**Fig. 2:**
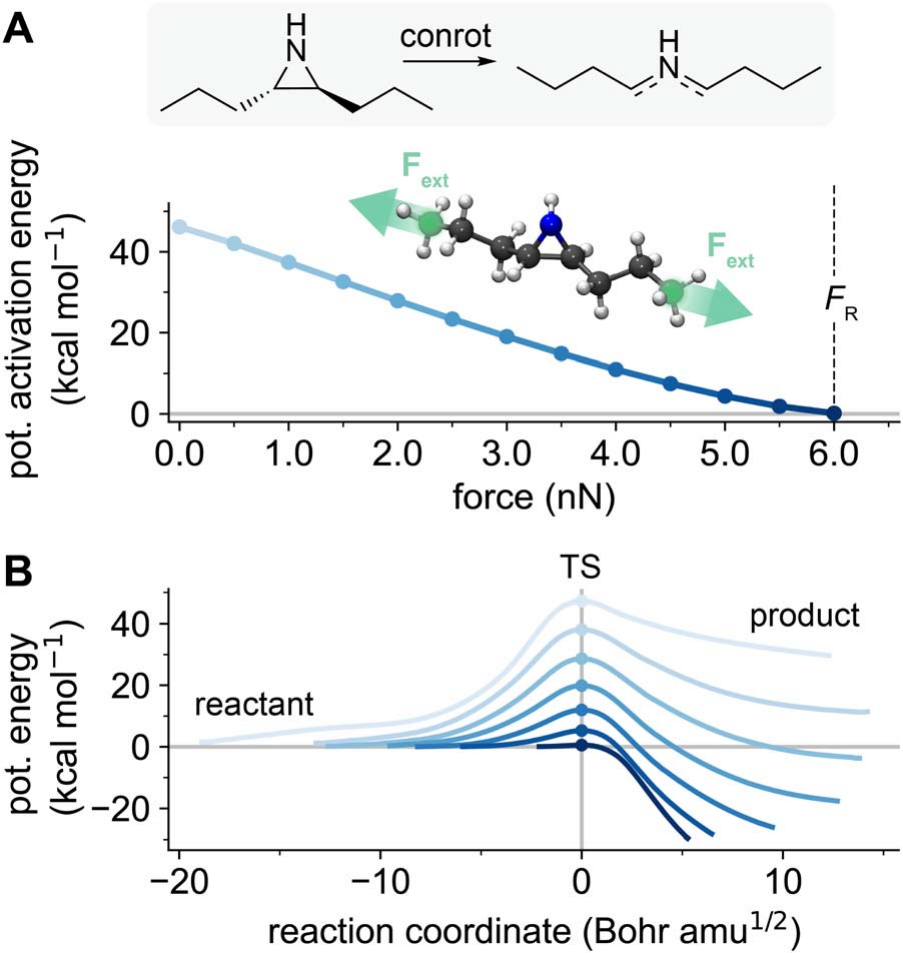
(A) Force-modified activation energy of the conrotatory ring-opening reactions of *trans*-dipropyl aziridine. The grey line marks 0 kcal mol^−1^, *i.e.* where the potential activation barrier vanishes, the dashed black line marks the rupture force *F*_R_, the magnitude of force where this occurs. The structure inlay shows the setup of the external pulling force applied during computations. Terminal carbon atoms to which the force is applied are marked in green. (B) Potential energies along the intrinsic reaction coordinates (IRCs) of the reaction, at integer values of external force up to *F*_R_ (0.0 nN to 6.0 nN). Increasing force is displayed in darker blue. Round markers denote the transition state structure, corresponding to the activation energies in (A). The grey line marks 0 kcal mol^−1^.

We used the FM-PES method to optimize force-modified transition state structures, reactants, and reaction pathways to study mechanochemical reactions.^8^Fig. 2A shows the decrease in potential activation energy by an external pulling force for a *trans*-dipropyl aziridine mechanophore. At 6.0 nN, the barrier is lowered to 0.2 kcal mol^−1^, and any further increase in external force would fully diminish it to 0 kcal mol^−1^. This marks 6.0 nN as the rupture force of this mechanophore. Past this magnitude of force, no transition state structure can be optimized.

Through the energy profiles along the force-modified intrinsic reaction coordinates (IRCs) shown in Fig. 2B, the rupture force catastrophe can be visualized: as the reaction barrier is continously lowered, the reactant minimum becomes increasingly shallow, until the potential is entirely flat. This coincides with the catastrophe-induced disappearance of the transition structure from the potential energy surface. As is evident from the barrier-force-relation, the mechanophore presented here is exceptionally well-behaved: the decrease in activation energy is close to linear, and adherence to the conrotatory ring-opening mechanism is strict. For such mechanophores, predicting reactivity and product stereochemistry is relatively straightforward. This changes when the complexity of the mechanophore system increases, and competitive reaction pathways complicate the picture. What differentiates a “complex” mechanophore from a “simple” mechanophore is not always evident from the structure. In the case of aziridine, the change from *trans*- to *cis*-substitution is enough to create a system with highly force-dependent competing mechanisms, which is much more challenging to study.

### The rupture force catastrophe of transition structures

In *cis*-dipropyl aziridine, the WH-favored conrotatory ring-opening reaction competes with the force-activated disrotatory reaction. Both mechanisms were studied through the optimization of force-modified stationary points and reaction pathways for external pulling forces ranging up to 4.0 nN. We found that the conrotatory and disrotatory reaction pathways are highly sensitive to the magnitude of external force. The transition structures corresponding to these reactions could only be optimized in a specific range of forces, outside of which it was not possible to converge to the correct structure (see Section S.1 in the SI for details on the optimization procedure). This is a common phenomenon in mechanochemical reactions, and has been noted before.^11,12,22,30–32^ Instead, optimizations converged to a transition structure corresponding to the respective other reaction mechanism once a threshold force was passed. This force-dependent (dis-)appearance of transition structures is reminiscent of the aforementioned rupture force catastrophe, an analogy we will further explore in the following.

**Fig. 3:**
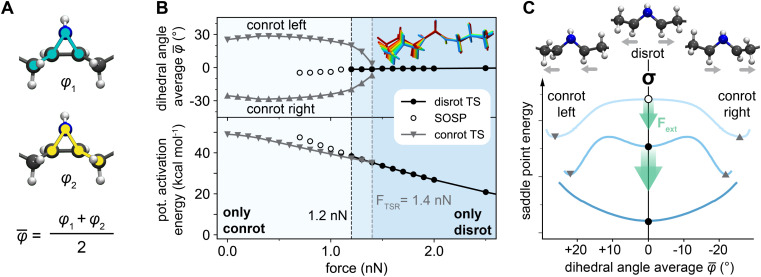
(A) Definition of the collective variable *φ̄* as the average of the two substituent rotation dihedrals. (B) Force-modified activation energies of the disrotatory and conrotatory ring-opening reactions (bottom) and dihedral angle average *φ̄* in the three transition state geometries at different forces (top). The structure inlay shows the geometry of the left-turning conrotatory transition structure for forces from 0.5 nN (blue) to 1.4 nN (red). (C) Effect of external force on aziridine ring-opening transition structures (TS), green arrows indicate the effect of the external force on the potential energy surface. Low force (light blue): two mirror-image conrotatory TS are separated by a disrotatory SOSP. Medium force (medium blue): conrotatory and disrotatory TS coexist as three first-order saddle points. High force (dark blue): conrotatory TS are no longer stationary points, only the disrotatory TS remains. The energetic profiles were obtained by performing restraint scans on a *cis*-dimethyl aziridine model compound. The details of this procedure are included in the SI.

The electronically favored conrotatory reaction path was found to be stable without external forces and in the low-force regime, up to a maximum force of 1.4 nN. Once the force exceeds this critical value, this reaction path becomes inaccessible. For the disrotatory pathway, an inverse behavior with regard to the external force was discovered: this reaction path only becomes available once the external force reaches 1.2 nN, instead being unavailable without and at low external forces. We found that the corresponding stationary point continues to exist on the potential energy surface as a SOSP in the force range where the mechanism is inaccessible, which is in agreement with discoveries made for similar systems in literature.^13,26^ The two energy-lowering modes of this SOSP are the mode associated with the disrotatory ring-opening motion, and an asymmetric twisting mode connecting the clockwise (“left”) and counterclockwise (“right”) conrotatory transition structures (see Fig. S7 in the SI). Movement along the second mode does not cause significant change in the end-to-end distance, indicating weak force coupling. The nature of these two modes was confirmed to be consistent across different forces and for all systems presented in this paper. For forces of a magnitude between 1.2 nN and 1.4 nN, conrotatory and disrotatory transition structures coexist, allowing the formation of multiple stereochemically distinct products. In Fig. 3B, the force-modified activation energies of both the conrotatory and disrotatory ring-opening reaction of *cis*-dipropyl aziridine are shown, and the critical forces after which first-order saddle points (transition structures) disappear are marked.

The selectivity control an external pulling force excerts on a reaction can be recognized by considering symmetry: the *cis*-substituted reactant is mirror-symmetric, as shown in Fig. 1. In the course of the conrotatory reaction, the mirror symmetry is broken by the substituent rotation, and the system's symmetry is lowered from *C*_S_ to *C*_1_. The disrotatory reaction on the other hand preserves the mirror plane, and maintains the *C*_S_ symmetry in the product. As the external force vectors have *C*_S_ symmetry, the symmetry-lowering conrotatory reaction is hindered by the force, as the required rotational motions run counter to the pulling direction.^8^ Despite the conrotatory reaction's atomic movements opposing the pulling direction, the activation energy is still lowered by the external force. The reason for this is that the ring-opening reaction consists of two distinct motions: only the conrotation of substituents is penalized by the external force, while the increase in the scissile bond's C–C distance is favored.

Past studies have shown that reaction pathways can become inaccessible when geometric constraints make the required atomic motions impossible.^25,26^ External pulling force has a similar restraining effect by energetically penalizing motions that run counter to the pulling direction. We investigated the geometries of the force-modified stationary points to understand this geometry-restraining effect in the force range close to the conrotatory pathway's disappearance. The average of the two dihedral angles *φ*_1_ and *φ*_2_ describing the linker rotation was selected as a measure of the effect of external force on mechanophore geometry. This collective variable *φ̄*, shown in Fig. 3A, was measured at the transition structures of both left- and right-turning conrotatory reactions and the disrotatory reaction for increasing forces. A *φ̄* of 0 results from a *C*_S_-symmetric structure, as is the case in the disrotatory transition structure. The absolute value of *φ̄* grows with increasingly asymmetric geometry. Fig. 3B shows that with increasing external force, *φ̄* of the conrotatory transition state structures approaches 0. This means that the asymmetric rotational motion required to proceed to the S-ylide product is becoming less pronounced in the transition structure as the force increases. Once the restraining effect of the force becomes too great, the conrotatory reaction path is eliminated and only the disrotatory reaction is available to the mechanophore. Fig. 3C illustrates how the deformation of the potential energy surface (PES) through an external force can result in the three qualitatively distinct scenarios marked as colored zones in Fig. 3B. The energetic profiles along this coordinate were computed through restraint scans at different forces using a *cis*-dimethyl aziridine model compound. A more detailed discussion of this procedure and the results of these scans is included in the SI, alongside the force-modified reaction barriers of the model compound. Here, we will use the energetic profiles qualitatively for the conceptualization of the observed disappearance of transition states.

Without external force, the Woodward–Hoffmann rules dictate that the conrotatory reaction takes place. At low magnitudes of external force (light blue in Fig. 3C), this mechanism persists as the magnitude of the force is not yet sufficient to circumvent the orbital symmetry rules and activate the disfavored mechanism. Two mirror-image conrotatory reaction pathways exist, one for each rotational direction, *i.e.* left- and right-turning. The disrotatory transition structure was found to be a SOSP with a mirror-symmetric structure, connecting the two conrotatory transition structures *via* its second energy-lowering mode. As the force increases (medium blue in Fig. 3C), the favorable symmetry relation between atomic motion and pulling force causes the disrotatory reaction path's activation energy to be lowered more strongly than that of the conrotatory reactions. This is evident from the slopes of the force-modified activation energies shown in Fig. 3B. Through this, the disrotatory SOSP is converted into a true transition structure, and the disrotatory reaction path becomes accessible. This describes the situation in the force range between 1.2 and 1.4 nN, where the conrotatory transition structures coexist with the disrotatory transition structure. When the force increases past 1.4 nN (dark blue in Fig. 3C), the barriers separating conrotatory and disrotatory transition structures disappear and the conrotatory reaction path becomes fully suppressed, and is thus inaccessible for the system. The conrotatory transition structures no longer exist as stationary points on the PES, which has turned into a slope.

Conceptually, this behavior is similar to the topological catastrophe leading to the rupture of a minimum structure^15^ when crossing the rupture force *F*_R_ as discussed above: transition structures “disappear” or “appear” when a particular value of the external force is crossed. In analogy we describe this behavior as transition state rupture, and named the corresponding force transition state rupture force, *F*_TSR_. For the *cis*-dipropyl aziridine system, *F*_TSR_ = 1.4 nN, as this is the maximum force at which the transition structure of the conrotatory reaction could be located.

### Effect of mechanophore core and linker substituents on transition state rupture

The transition state rupture force *F*_TSR_ is strongly influenced both by the linker substituents acting as force-transferring levers and by the intrinsic properties of the mechanophore core itself. Three different 4π-electron mechanophores, cyclobutene, aziridine and oxirane, were selected for this study. Through the choice of mechanophore, the degree of adherence to the classical Woodward–Hoffmann reactivity can be controlled. An approximate measure of this was determined by computing the diradical character for the *cis*-dipropyl substituted mechanophores along the IRC pathway of the WH-favored conrotatory ring-opening reaction without external force. The exact procedure used to determine the diradical character and an in-depth discussion of the results can be found in the SI.

**Fig. 4:**
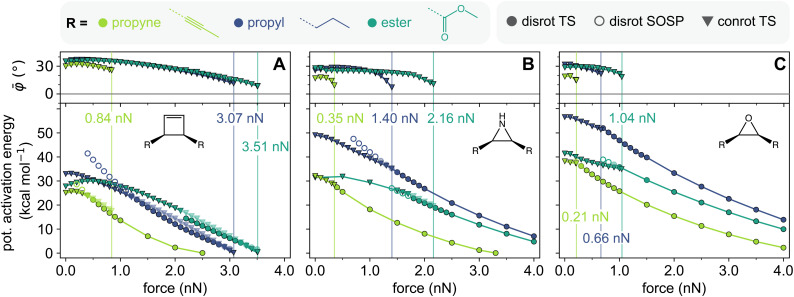
Force-modified activation energies for the conrotatory and disrotatory ring-opening reactions of cyclobutene (A), aziridine (B), and oxirane (C), and dihedral angle average *φ̄* of the conrotatory transition structures. All mechanophores were *cis*-substituted with propyl (blue), ester (teal), and propyne (green) linkers. The connection between linker and mechanophore is shown as a dashed line in the linker structures. Transparent markers denote the higher energy mechanism in force ranges where two mechanisms compete. Vertical lines mark the transition state rupture force *F*_TSR_ of the conrotatory ring-opening.

From cyclobutene to aziridine to oxirane, the diradical character increases both in the transition structure and product, as summarized in Table 1. The diradical nature of the conrotatory ring-opening reaction can be seen as a predictor for the magnitude of *F*_TSR_. In Fig. 4, the force-modified activation energies of the conrotatory and disrotatory ring-opening reactions of the three mechanophores with varying linker substituents are shown, and *F*_TSR_ is marked for each of the studied systems. Regardless of the linker substituent, *F*_TSR_ is always greatest in the cyclobutene mechanophore, followed by aziridine. The oxirane mechanophore consistently has the smallest *F*_TSR_, *i.e.* the change from conrotatory to disrotatory ring-opening mechanism occurs at the lowest value of external force for all three mechanophores studied here. The conrotatory reaction mechanism of this mechanophore already has a large contribution of diradical electronic structure and therefore, the electron pair is already partially broken, enabling a disrotatory reaction path which is known to pass through an almost purely diradical transition structure.^8,9,12,33^ Effectively, this makes the conrotatory pathway more susceptible to the geometric deformation imposed by the external forces, and less force is required to eliminate the conrotatory pathway. We can therefore conclude that the higher the diradical character of the WH-allowed reaction, the less the reaction will proceed through a conrotatory mechanism when external force is applied.

**Table 1:** Computed diradical character at the transition structure and product geometry of the conrotatory reaction without external force for the three studied mechanophore cores with *cis*-propyl linkers

Mechanophore	Diradical character (%)	
	Transition structure	Product
Cyclobutene	11	5
Aziridine	31	14
Oxirane	66	34

In addition to the effect of the mechanophore core, different linkers were studied in order to show the importance of the force-transferring substituents for stereochemical selectivity. The three linkers used in this study are propyl, propyne, and methyl ester. They were selected based on the hypothesis that *F*_TSR_ behaves similarly to the rupture force of mechanophore reactants, *F*_R_. In a previous computational study that systematically compared the effect of linker substituents on the reactant rupture force of various mechanophores, propyne linkers and ester linkers have been shown to have the strongest effect on the force-sensitivity of molecules.^34^ Additionally, ester linkers are commonly used in mechanophore activation experiments.^20,35^ Propyne substituents drastically destabilize mechanophores by lowering their rupture force, while ester linkers cause an increase in rupture force.^34^ A more recent model for the prediction of linker effects further corroborates this finding:^36^ propyne linkers are highly efficient in transferring force to mechanophores, comparable to phenyl linkers, which are known from experiments to significantly increase mechanochemical susceptibility.^37,38^ This effect is related to the angle of the linker atoms directly adjacent to the scissile bond, which control the transduction of force to the mechanophore.^34,36^ Angled linkers, like *Z*-alkenes, show poor force-transferring properties, while linear linkers are strongly activating.^36^

We found that this trend is consistent also for the transition state rupture events: all mechanophores show a very low *F*_TSR_ when propyne linkers are used to transfer force to the scissile bond. The propyne-substituted mechanophores all switch to the disrotatory ring-opening mechanism at less than 1 nN external force. The reason for this strong effect is the rigidity of the linker: as there are less degrees of freedom through which the force-imposed geometric strain can be compensated, lower forces are needed to induce a critical deformation of the conrotatory transition structure. On the other hand, ester-substituted mechanophores have the greatest *F*_TSR_ values, which is in further agreement with the analogy between *F*_R_ and *F*_TSR_. The propyl substituents consistently results in an *F*_TSR_ value in the intermediary range between the extremes of the propyne and ester linker. In the case of the cyclobutene mechanophore, the conrotatory ring-opening mechanism persists up to the reactant's rupture force for propyl and ester linkers, meaning that for these systems no transition state rupture event takes place.

When considering the electronic effect of the linkers on the WH-favored reaction, propyne and ester substituents are remarkably similar: without external force, the activation energies of the conrotatory reactions with these two substituents differ by 3 kcal mol^−1^ or less for all three mechanophore systems. This is interesting when considering their inverted mechanistic stereoselectivity, *i.e.* a strong favoring of the dis-rotatory or conrotatory reaction for propyne and ester linkers respectively, which seems to result from geometric rather than electronic effects.

While the here presented study focuses exclusively on 4π-electron mechanophores, experimental observations made for the 2π-mechanophore dichlorocyclopropane (DCP) indicate that the effect of the linker on the ring-opening mechanism might be applicable to force-induced electrocyclic reactions on a more general basis:^39^ for *trans*-substituted DCP, ester linkers cause the ring-opening reaction to follow the electronically favored but force-hindered disrotatory pathway, contrary to the expected activation of the force-supported conrotatory reaction as known from the alkyl-substituted mechanophore. This provides a highly interesting avenue for future studies that could expand the transition state rupture concept to mechanophores beyond those presented here. Currently, the possibility that the linker substituent can qualitatively affect the reaction mechanism is rarely considered. In the following section, we will show the full extent of this implication by applying the concept of transition state rupture to two experimental studies of aziridine mechanophores.^20,21^

### Explaining aziridine mechanochemistry

The results discussed in the previous section showed that *cis*-oxirane has a strong tendency to undergo disrotatory ring-opening under force, making the W-shaped carbonyl ylide the primary reaction product. As this ylide is only meta-stable, it closes to the *trans*-reactant *via* the WH-favored pathway once the force recedes. Thus, the mechanochemical ring-opening becomes an observable isomerization from *cis*- to *trans*-oxirane, consistent with reported experiments.^40,41^ The barrier height for the force-free ring-closing from W-shaped carbonyl ylide to *trans*-oxirane is 10.0 kcal mol^−1^, as reported in a previously published study.^13^

In the experimental study of *N*-methoxybenzene aziridine,^20^ two observations have been made after sonication: a lack of isomerization from *cis* to *trans* and *vice versa*, and 1,3-cycloaddition with DMAD resulting in adducts stereochemically inverse to the reactant, *i.e. cis*-substituted aziridine to *trans*-adduct and *trans*-substituted aziridine to *cis*-adduct. The authors postulated an ylide-free mechanism based on comparison with the mechanochemical behavior of oxirane.^40,41^ However, both of their findings are consistent with the formation of an S-shaped ylide through conrotatory ring-opening of the *cis*-substituted mechanophore, a theory surprisingly ignored in the original publication:^20^ In this case, the thermal ring-closing of the ylide would re-form *cis*-aziridine and no observable change in reactant stereochemistry would occur. In addition, the resulting S-shaped azomethine ylides form *trans*-adducts with DMAD, which would further confirm the experiments.^17,18^ For *trans*-substituted aziridine, the conrotatory reaction is without competition, as previously discussed. The W-shaped ylide product of this reaction forms a *cis*-configured DMAD adduct, matching experimental observations.^18^ In the experimental study of Jung and Yoon, ester linkers are used as force-transferring substituents,^20^ which are shown to cause the conrotatory reaction mechanism to persist to higher forces (see Fig. 4).

**Fig. 5:**
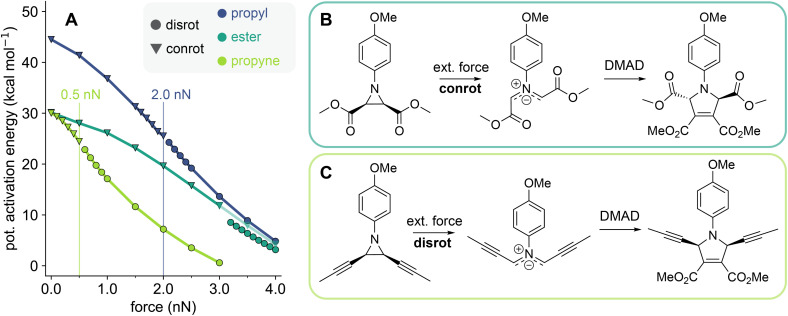
(A) Force-modified activation energies of the conrotatory and disrotatory ring-opening reactions of *N*-methoxybenzene aziridine *cis*-substituted with propyl (blue), ester (teal, experimentally studied system^20^), and propyne (green) linkers. Transparent markers denote the higher energy mechanism in force ranges where two mechanisms coexist. Vertical lines mark *F*_TSR_. (B) Reaction mechanism of the force-activated conrotatory ring-opening reaction of *cis*-diester substituted *N*-methoxybenzene aziridine and subsequent addition of DMAD, experimentally studied system.^20^ (C) Reaction mechanism of the force-activated disrotatory ring-opening reaction of *cis*-dipropyne substituted *N*-methoxybenzene aziridine and subsequent addition of DMAD, computational prediction.

To investigate whether the TS-rupture force of the conrotatory reaction is indeed greater than the magnitude of force necessary for ring opening, we have computed transition structures and activation energies for both the con- and dis-rotatory ring-opening reactions of the experimentally studied *cis*-diester *N*-methoxybenzene aziridine (see Fig. 5). Additionally, propyl and propyne linkers were investigated to compare the influence of the linker on the respective transition state rupture forces. In order to properly replicate the experiments, which have been performed in THF solvent, a polarizable continuum implicit solvation model was used.^20,42^ Our computations, presented in Fig. 5A, show that the *N*-substituent generally causes an increase in *F*_TSR_ in comparison to the plain aziridine mechanophore. This is true for all studied linkers. For the diester-substituted system, the conrotatory reaction path is remarkably persistant and no transition state rupture event was found in the studied force range from 0 to 4 nN. Furthermore, the conrotatory ring-opening reaction is the only available mechanism up to an external force of 3.2 nN.

It is difficult to determine the exact magnitude of the force required to activate a mechanophore under ultrasound conditions. The collapse of microbubbles during sonication is known to create localized hot-spots, with temperatures in the solvent shell surrounding a cavitation event reaching up to 2000 K.^43,44^ With local temperatures at the mechanophore impossible to determine, we can only estimate how far activation energies need to be lowered for a mechanochemical reaction to occur. At room temperature (298.15 K), reaction barriers of 10 kcal mol^−1^ have a half-life of approximately 1.5 μs, which is well within the timescale of the pulling forces induced during sonication.^45–47^ While this marks a lower bound for reaction barriers where mechanochemical activation can be expected, the aforementioned temperature fluctuations can allow higher barriers to be overcome. The barrier of the conrotatory ring-opening reaction at 2.5 nN external force was computed to be 15.8 kcal mol^−1^. As the disrotatory pathway could only be found at forces of 3.2 nN and higher, this strongly supports our hypothesis of a conrotatory mechanism dominating the reactivity in the sonication-activated reaction, leading to an S-shaped ylide. When propyl linkers are used, however, *F*_TSR_ is lowered to 2.0 nN, causing a switch to the disrotatory ring-opening reaction earlier, in agreement with the trend observed in the previous section. This also holds true when considering propyne linkers for the mechanochemical ring opening of *N*-methoxybenzene aziridine, where the conrotatory reaction becomes unavailable at forces greater than only 0.5 nN. As the propyne linkers also improve the force-responsiveness of the mechanophore, the computationally predicted activation energies are much lower than in the diester system. We hence propose that by changing the force-transferring linkers from ester to propyne and repeating the sonication experiments, observations matching the formation of a W-shaped azomethine ylide *via* disrotatory reaction should be made, *i.e.* the formation of a the *cis*-configured adduct when adding DMAD or the isomerization from *cis*- to *trans*-aziridine.

**Fig. 6:**
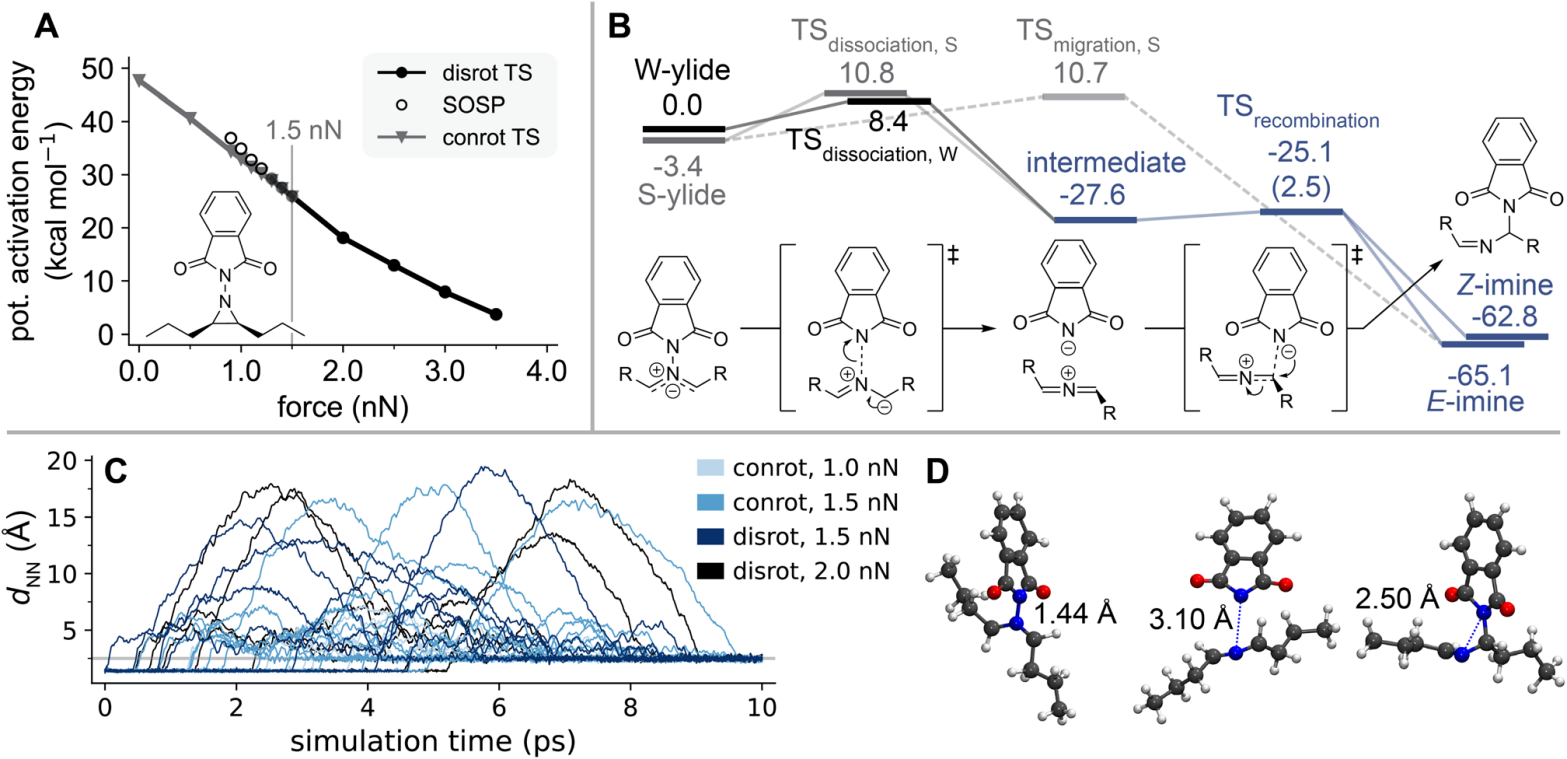
(A) Force-modified activation energies of the disrotatory and conrotatory ring-opening reactions of *cis*-dipropyl *N*-phthalimido aziridine. The vertical line marks *F*_TSR_. (B) Potential energy profile of the S-ylide's (grey) and W-ylide's (black) phthalimide migration reaction. The part of the reaction pathway shared between S- and W-ylide is depicted in blue. The reaction mechanism of the W-ylide to Z-imine reaction is shown as an example, other reactions proceed likewise. All energies are listed in kcal mol^−1^ and were computed without external force modification. (C) Change in N–N distance *d*_NN_ during the AISMD simulations started from the con- and disrotatory transition structures of *cis*-dipropyl-*N*-phthalimido aziridine at different forces. Only productive trajectories are shown, a full discussion of AISMD results is included in the SI. The grey line marks 2.5 Å, the average *d*_NN_ in the imine product. (D) Snapshots from an AISMD trajectory at 1.0 nN external force, showing the ylide structure, the intermediate formed after dissociation of the N–N bond, and the imine product, with *d*_NN_ at each structure.

In another paper, the mechanochemical ring-opening of *cis*-dipropyl *N*-phthalimido aziridine was reported, which was accompanied by a 1,2-migration of the phthalimide substituent, resulting in a hydrolyzable imine.^21^ We have found that the force-dependent mechanism switch from conrotatory to disrotatory ring-opening reaction also occurs in the experimentally studied system using propyl linkers.^21^ The computed activation energies of these reactions are shown in Fig. 6A. In both the con- and disrotatory reaction, a stable ylidic intermediate is formed, with a diradical character of approximately 21% as calculated from the effective number of unpaired electrons (ENUE, see Section S.6 of the SI) without external force modification.^48^ For the system discussed here, the conrotatory reaction path becomes inaccessible past an external force of 1.5 nN. Between 1.3 nN and 1.5 nN, both dis- and conrotatory reaction pathways coexist with a difference between the respective activation energies of less than 0.5 kcal mol^−1^. To gain a complete picture of the post-ring-opening reactivity of *N*-phthalimido aziridine, the follow-up chemistry of both S- and W-ylide was explored.

Fig. 6B shows the computed potential energy profile of the migration reactions starting from the S- and W-ylide. The energies of the pathways are given relative to the W-ylide's energy. No external force was included in the computations, as these follow-up reactions showed very little force-dependence (see Fig. S17 in the SI). For both ylides, the migration reaction can occur as a two-step process: the first step in this reactions is the dissociation of the N–N bond, resulting in the formation of a linear azaallenium cation and a phthalimide anion. The heterolytic nature of this cleavage was verified by an ENUE of 0.39 at the transition structure of the W-ylide reaction, corresponding to approximately 20% diradical character. With low activation energies of 8.4 kcal mol^−1^ and 14.1 kcal mol^−1^ for the W- and S-ylide respectively, this reaction should occur in quick succession to the initial ring-opening reaction. The two ionic fragments recombine in an almost barrierless process (2.5 kcal mol^−1^ potential activation energy) through nucleophilic attack of the anionic nitrogen at a carbon atom neighboring the azaallenium nitrogen, resulting in the imine product. Depending on the site of this attack, an *E*- or *Z*-configured imine can be formed, with the *E*-isomer being slightly more stable. In addition to this two-step migration mechanism, a direct pathway from the S-ylide to the *E*-configured imine was found. The activation energy of this reaction is almost equal to that of the dissociation reaction. Due to steric hindrance, the concerted pathway was not found for the W-ylide.

To capture post-transition state dynamic behavior, *ab initio* steered molecular dynamics (AISMD) simulations were used to further investigate these reaction pathways.^49,50^ Trajectories were initialized from the ring-opening transition structures at different magnitudes of external force, proceeding towards the ylide product. Spherical boundary conditions (SBC) in form of a bias potential1
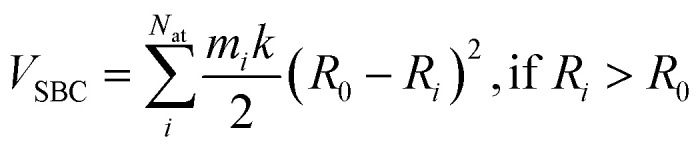
have been applied to prevent diffusion of the intermediate fragments and mimick the effect of a solvation cage: here, *N*_at_ is the number of atoms of the system, *k* is the corresponding force constant and *m*_*i*_ and *R*_*i*_ are mass and distance from the center of mass of mass of atom *i*, respectively. *R*_0_ is the size of the confining potential emulating the solvation cage and has been chosen to 10 Å. A detailed discussion of this AISMD setup can be found in the SI.

The distance between the two nitrogen atoms, denoted as *d*_NN_, was chosen as a parameter to track the migration reaction progress during the simulations, shown in Fig. 6C. In the ylide, which is present at the start of all trajectories, *d*_NN_ ≈ 1.44 Å on average. In the simulations, we observed a rapid increase in *d*_NN_, resulting from the dissociation of the N–N bond. The two fragments, though oppositely charged, do not recombine immediately, often remaining separate for several picoseconds. During this time, *d*_NN_ varies greatly as the molecules freely diffuse within the space confined by the SBC. Recombination of the azaallenium/phthalimide fragments to the imine product known from experiment was observed when they encounter each other in a favorable geometry, which is hindered by a translational entropic barrier. In the imine product, *d*_NN_ ≈ 2.50 Å, marked by a grey line in Fig. 6C. The formation of both *E*- and *Z*-configured imines was observed in the dynamics simulations. The two-step migration mechanism was found regardless of the magnitude of external force, or whether the trajectory originated from the con- or disrotatory transition state. Concerted migration reactions were not observed in the simulations, showing this mechanism to be dynamically disfavored despite its low activation energy. As the second step of the dissociation/recombination pathway is identical for the S- and W-ylide, it is not possible to trace back to the nature of the ring-opening reaction from the migration reaction product. Given the similar reaction barriers of the conrotatory and disrotatory pathways, it appears likely that both reactions take place. The migration reaction proceeds in two steps from both S- and W-ylide structures, leading to the hydrolyzable imine product that was found after sonication. As the migration of the phthalimide substituent involves an ionic intermediate state, the solvent polarity should have a strong influence on the reaction, which would be an interesting avenue for further experimental study of the *N*-phthalimido aziridine mechanophore.

## Conclusion

Our investigations of mechanochemical ring-opening reactions could show that through the magnitude of external force, a mechanistic switch between con- and disrotatory ring-opening reactions can be triggered. Through the deformation of molecular geometry, reaction mechanisms are either enabled or suppressed, depending on whether the external force supports or hinders the reaction motion. The symmetry-disfavored disrotatory reaction path becomes available to the mechanophore only once the external force exceeds a certain magnitude. At forces insufficient for the activation of this pathway, the dis-rotatory transition structure was found to be a second order saddle point on the potential energy surface, which is transformed into a true transition structure through the pulling force. The conrotatory transition structure, however, is eliminated in a transition state rupture event at forces exceeding a critical value, leading to a switch in the ring-opening mechanism from conrotatory to disrotatory in a system-specific force range. We could show that both the mechanophores' intrinsic properties as well as the force-transferring substituents can be used to tune the transition state rupture force *F*_TSR_: the diradical character of the WH-favored reaction can be used as a predictor for a mechanophore's susceptibility to transition state rupture events. Higher diradical character during that reaction results in lower *F*_TSR_ as deviation from Woodward–Hoffmann-reactivity is facilitated. While propyne linkers consistently caused the conrotatory pathway to become inaccessible at forces less than 1.0 nN, ester linkers had a profound stabilizing effect on this mechanism, in some cases preventing transition state rupture events entirely. This shows that linker substituents can dramatically influence a mechanophore in ways that go far beyond simply tuning reaction barriers. Indeed, through the choice of linker, product stereochemistry can be influenced by controlling whether the conrotatory or disrotatory ring-opening mechanism is activated.

Using the concept of transition state rupture we could derive an explanation for the hitherto unanswered questions of aziridine mechanochemistry and elucidate the reaction mechanisms of the mechanochemical ring-opening of two prior experimental studies.^20,21^ In particular, our computations show that the ester linker stabilizes the conrotatory ring-opening mechanism of *N*-methoxybenzene aziridine, making it dominant thoughout all force regimes. The reactivity of the S-ylide intermediate is in agreement with the experimental observations. Additionally, the mechanism of the *N*-migration observed upon activating *N*-phthalimido aziridine was revealed through molecular dynamics simulations, showing how the mechanochemical imine formation proceeds as a multi-step reaction. These exemplary cases highlight the complexity and importance of distinguishing thermal and mechanochemical reaction mechanisms. Many factors influence the competition behavior between force-modified pathways, and in some situations the seemingly unfavorable reaction can still dominate reactivity.

## Author contributions

Anne Germann: conceptualization, methodology, validation, investigation, formal analysis, writing – original draft, visualization. Jan Meisner: conceptualization, methodology, validation, resources, writing – review & editing, supervision, project administration, funding acquisition.

## Conflicts of interest

There are no conflicts to declare.

## Supplementary Material

SC-016-D5SC04954G-s001

## Data Availability

Data for this article, including molecular geometries and movies of exemplary AISMD trajectores are available at Zenodo at https://doi.org/10.5281/zenodo.16758919. Supplementary information containing computational details, potential energies, and additional computational results. See DOI: https://doi.org/10.1039/d5sc04954g.
