# Supplementary material for: Force-induced transition state rupture enables mechanistic control in aziridine mechanochemistry
Source: Chem Sci. 2025 Aug 16;16(45):21454–63. doi: 10.1039/d5sc04954g (PMC12371473; doi:10.1039/d5sc04954g)
Supplement: SC-016-D5SC04954G-s001 [file SC-016-D5SC04954G-s001.pdf]

Supplementary Information (SI) for Chemical Science.
This journal is © The Royal Society of Chemistry 2025

Supporting Information

Force-induced Transition State Rupture Enables Mechanistic Control in Aziridine Mechanochemistry

Anne Germann[a] and Jan Meisner[a]*
[a]Institute for Physical Chemistry, Heinrich Heine University Düsseldorf
Universitätsstraße 1, 40225 Düsseldorf, Germany
*Email: meisner@hhu.de

S.1 Optimization of force-modified stationary points and minimum energy pathways

For all optimizations of stationary points and intrinsic reaction coordinates (IRCs), the DL-FIND algorithm library was used.[1] Minimum energy geometries were optimized using the L-BFGS algorithm[2], transition structures (TS, first order saddle points) as well as second order saddle points (SOSPs) were obtained with the dimer method.[3,4] While the dimer method is an algorithm developed for the optimization of first order saddle points, SOSPs could be located by decreasing the external force in small steps, effectively trapping the optimizer on a ridge where the nearest stationary structure is the desired SOSP. DL-FIND was interfaced to TeraChem[5–7] and Molpro[8–10] *via* ChemShell.[11] To investigate the behavior of molecules under external forces, the force-modified potential energy surface (FM-PES) approach was used, assuming isotensile conditions, i.e. constant force on the time scale of molecular motion.[12] The forces are applied to the outermost carbon atoms adaptively, such that the orientation of the molecule in space does not affect the total energy of the system, which is equivalent to the *external force explicitly included* (EFEI) method.[13] The external force was included in the computations through a modification of the potential and implemented in a modified version of the ChemShell (tcl). Here, the effective force-modified potential is calculated as the sum of the electronic *ab initio* potential and the mechanical force term, $-F \cdot \Delta x$, where F is the external force and Δx is the distance between the two atoms to which force is applied (see Figure S1).[12,13]

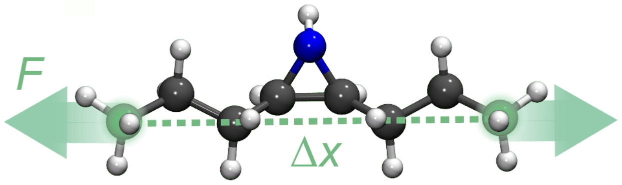

Figure S1: Setup of the external forces applied during computations. Terminal carbon atoms to which the force is applied are marked in green.

Minimum structures were confirmed by the absence of negative eigenvalues of the Hessian matrix. All transition state structures were confirmed to be first order saddle points through the presence of a single negative eigenvalue of the Hessian matrix. SOSPs were characterized by the presence of two negative eigenvalues of the Hessian matrix. Stationary points were optimized up to a maximal component of the nuclear gradient smaller than $4.5 \cdot 10^{-5}$ atomic units, with the exception of second-order saddle points, for which this criterion had to be loosened up to $8.0 \cdot 10^{-4}$ atomic units at lower forces. To ensure the correct SOSP was optimized in all cases despite this, the mode corresponding to the second imaginary frequency was rigorously checked for each structure. Only those SOSPs displaying a mode that could be identified unambiguously as the correct symmetry-breaking mode were included in this work.

DFT calculations with a symmetry-broken set of guess orbitals were used to find and optimize structures with a diradical character.[14] The TeraChem quantum chemistry package was used with one GPU.[5–7] All DFT computations were done using the 6-31G* basis set[15–17] and the B3LYP functional[18–21] with D3 dispersion correction included.[22] The DFT grid contains 3000 points per atom. Pulay's DIIS algorithm was used for SCF convergence[23], with the largest component of the DIIS error vector converged to $1.0 \cdot 10^{-6}$.

For all energies presented in the main text, the accuracy of the computed activation energies was improved by performing single-point energy computations at the complete active space perturbation theory of second-order (CASPT2)[24] level of theory using the cc-pVTZ basis set[25] on DFT geometries. This particularly improves the description of static correlation, which is important for the correct description of structures with a (partial) diradical character. Other works have shown that the geometries optimized at the DFT level are sufficiently accurate to justify their use.[26–28] All CASPT2 computations were performed using the Molpro 2022.3 quantum chemistry program package.[8–10,29–36] The procedure used for the selection of the active space for all systems is detailed in the next section. All additional energies reported in this Supporting Information document were computated at the B3LYP+D3/6-31G* level of theory.

The geometries of the systems derived from experimentally studied mechanophores *N*-methoxybenzene aziridine and *N*-phthalimido aziridine were optimized using the Polarizable Continuum Model (PCM) variant of the Conductor-Like Solvation Model (COSMO)[37–39] with a dielectric constant $\epsilon = 7.58$[40] to meet the experimental conditions of THF solvent. As no implicit solvation is available for CASPT2 computations in Molpro 2022.3, we chose to add the potential energy contribution from the solvation in the DFT computation to the CASPT2 energy.

$$V_{\text{CASPT2}}(\text{THF}) = V_{\text{CASPT2}}(\text{vacuum}) + (V_{\text{DFT}}(\text{THF}) - V_{\text{DFT}}(\text{vacuum}))$$

S.2 Active space orbitals used in CASSCF and CASPT2 computations

To determine the ideal active space for the multiconfiguration computations of each system, a complete active space (CASSCF)[41] single-point computation with a large active space ((8e,8o) to (16e,16o), depending on the system) was performed. Only those orbitals that deviated sufficiently from being fully

occupied or empty, i.e. with occupations < 1.95 or > 0.05, were included in the active space used to obtain energies presented in this paper. This initial orbital scan was performed on a force-modified transition state structure of the disrotatory ring-opening reaction. For the *N*-phthalimido ylide follow-up reactions, the transition structure of the dissociation reaction was used. The optimized active space of each studied system is shown in the following Figures S2, S3, and S4.

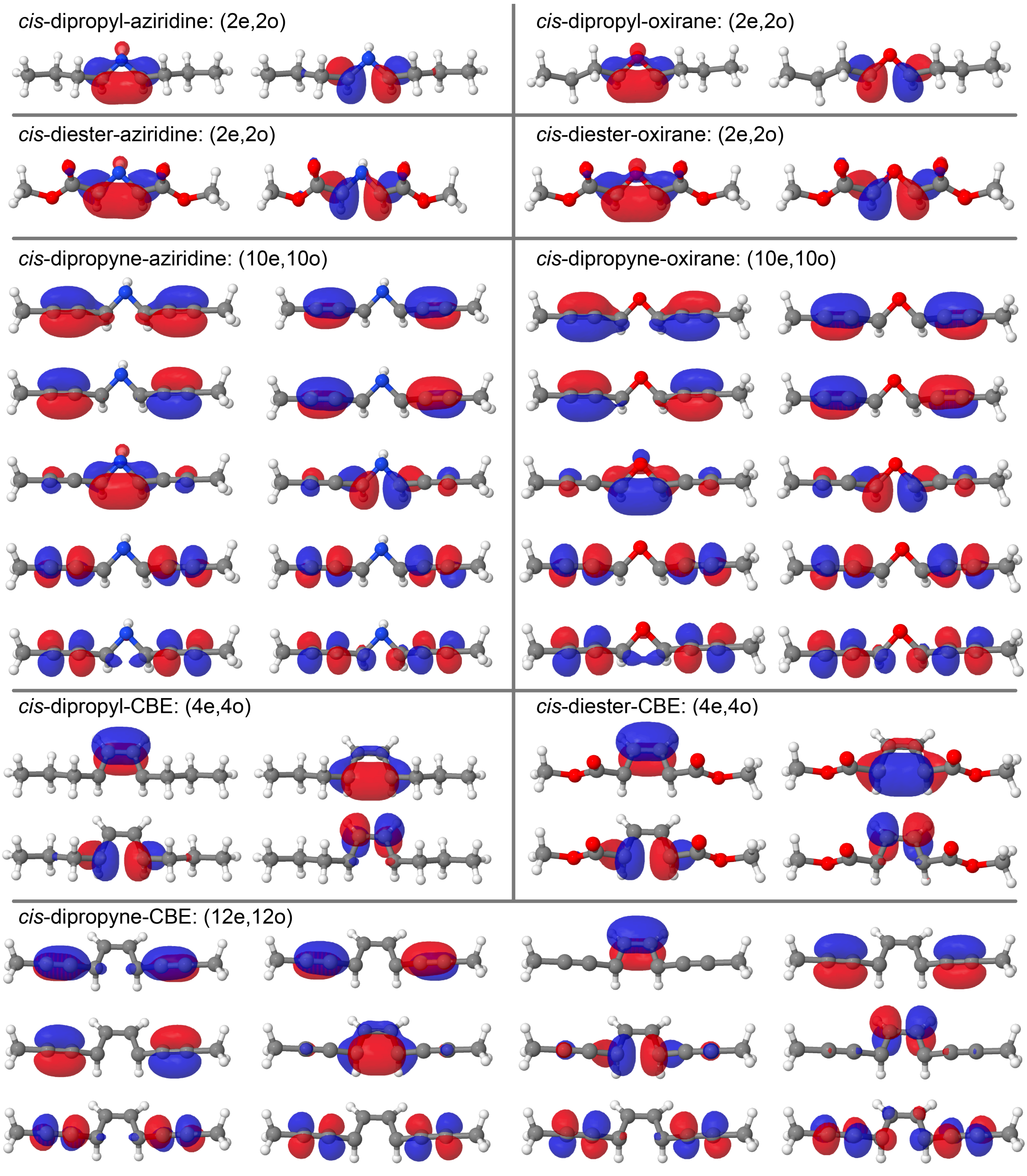

Figure S2: Active space orbitals at the disrotatory ring-opening transition structure.

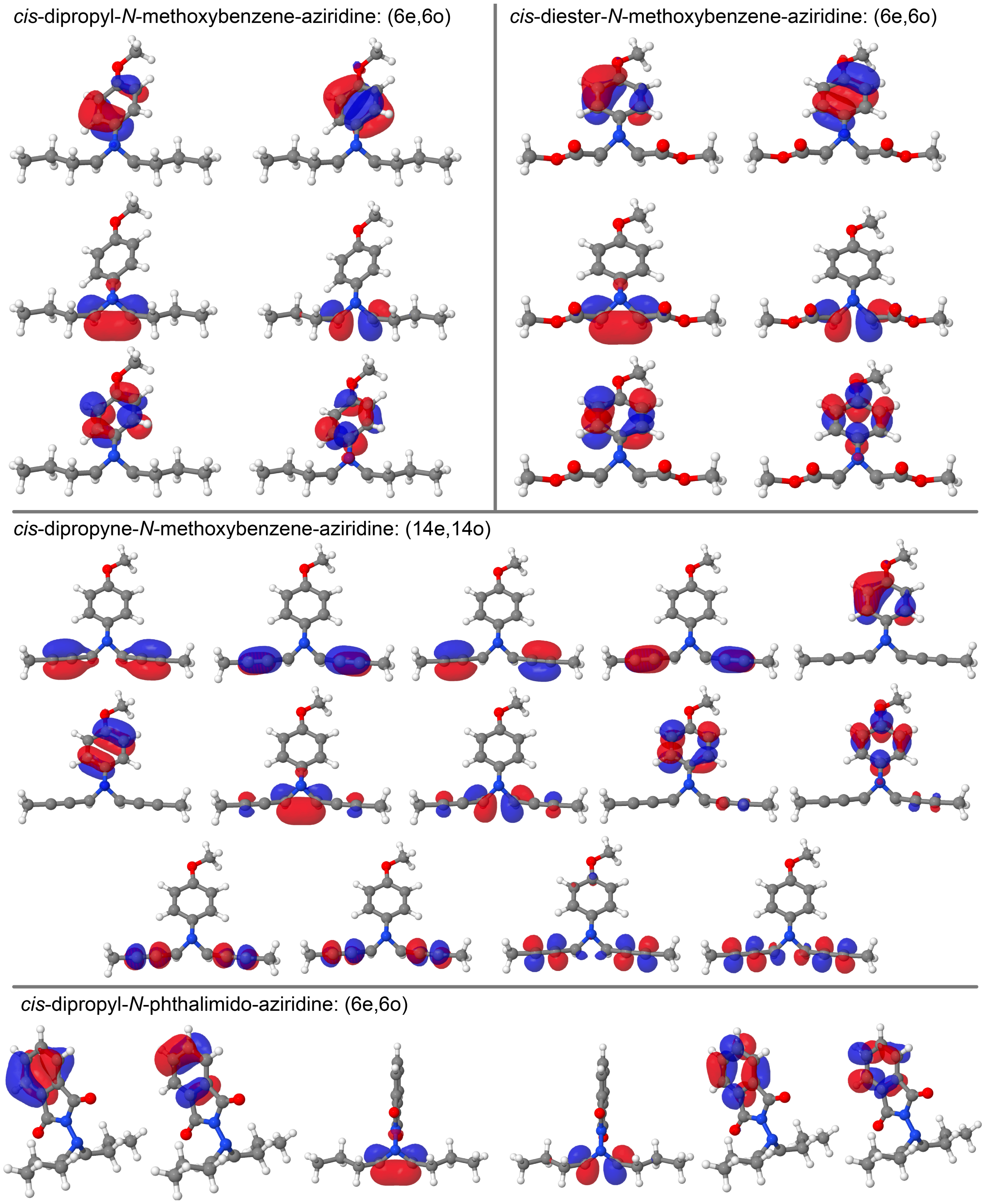

Figure S3: Active space orbitals at the disrotatory ring-opening transition structure (Cont'd).

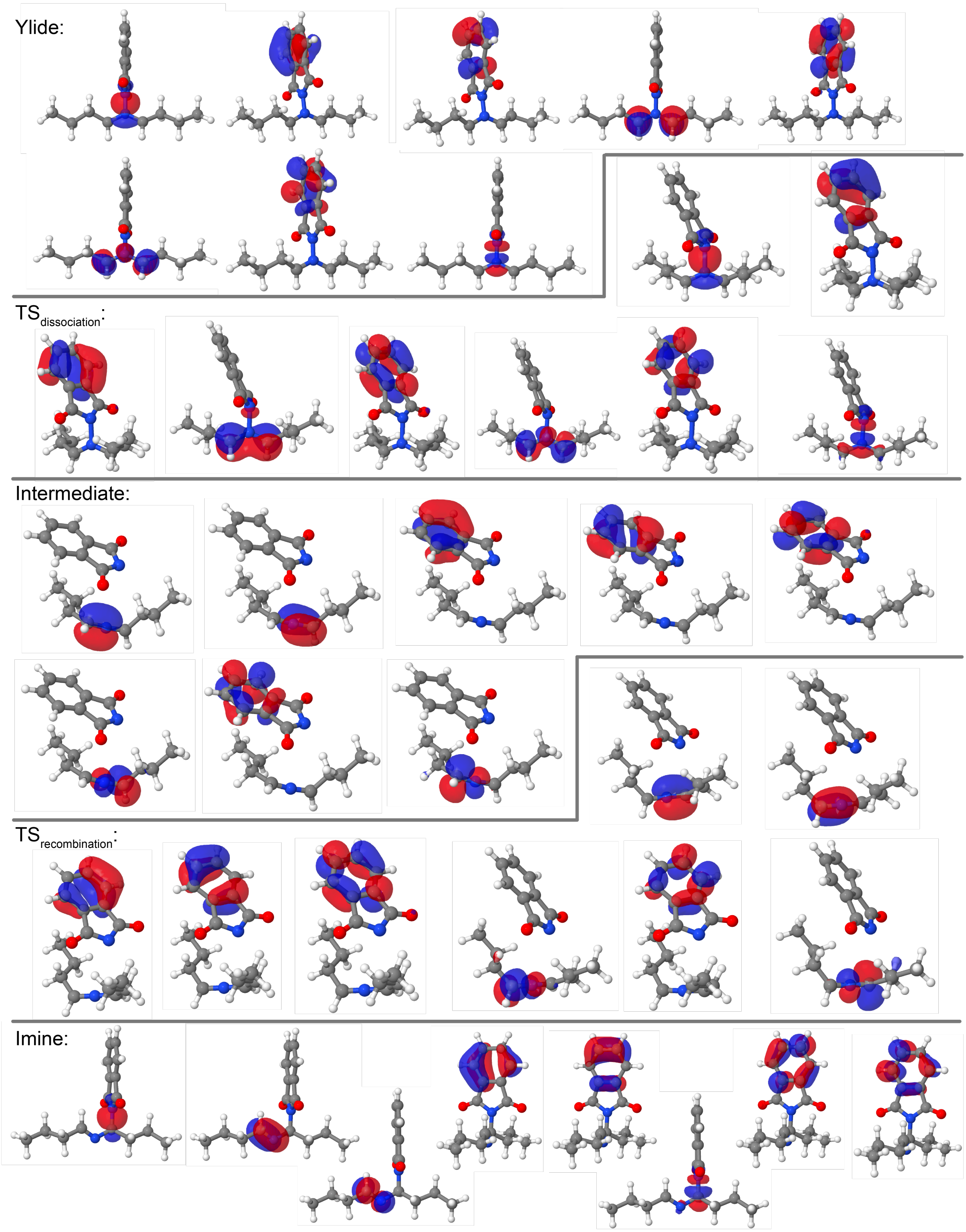

Figure S4: Active space orbitals used for the investigation of *N*-phthalimido ylide follow-up reactions.

S.3 Restraint scans along the symmetry-breaking coordinate of the *cis*-dimethyl aziridine model compound

To visualize the dimension of the force-modified potential energy surface on which the conrotatory and disrotatory transition states are related by symmetry, restraint transition state optimizations were performed using *cis*-dimethyl aziridine. This structure was chosen as an idealized model compound, as the small linkers result in geometries of higher symmetry. For the dipropyl-substituted aziridine used for the discussion of activation energies in the main text, surplus degrees of freedom on the linker lower the symmetry and increase the noise in the energetic profiles. For these scans, one of the two dihedral angles, φ_1, was restrained and all other geometry parameters, including φ_2, were allowed to relax during the optimizations. Figure S5 shows the relation between the restraint parameter φ_1 and the relaxed parameters φ_2 and $\bar{\varphi}$. From the restrained transition state structures, $\bar{\varphi}$ was computed and plotted against the relative potential energy. Profiles were computed for positive values of $\bar{\varphi}$, then mirrored to obtain the full range. In the resulting force-modified energy profiles, minima represent transition structures and maxima represent higher order saddle points. The first restraint scan was initiated from the disrotatory transition structure at 1.0 nN, proceeding stepwise towards the conrotatory transition structure by decreasing φ_1. Once the full 1.0 nN profile was optimized, all structures were reoptimized at 0.5 nN and 1.5 nN respectively to obtain the low- and high-force FM-PES slices. The 0.5 nN scan was then extended by the same stepwise optimization procedure, as the range was not yet sufficient to fully map the conrotatory transition structure.

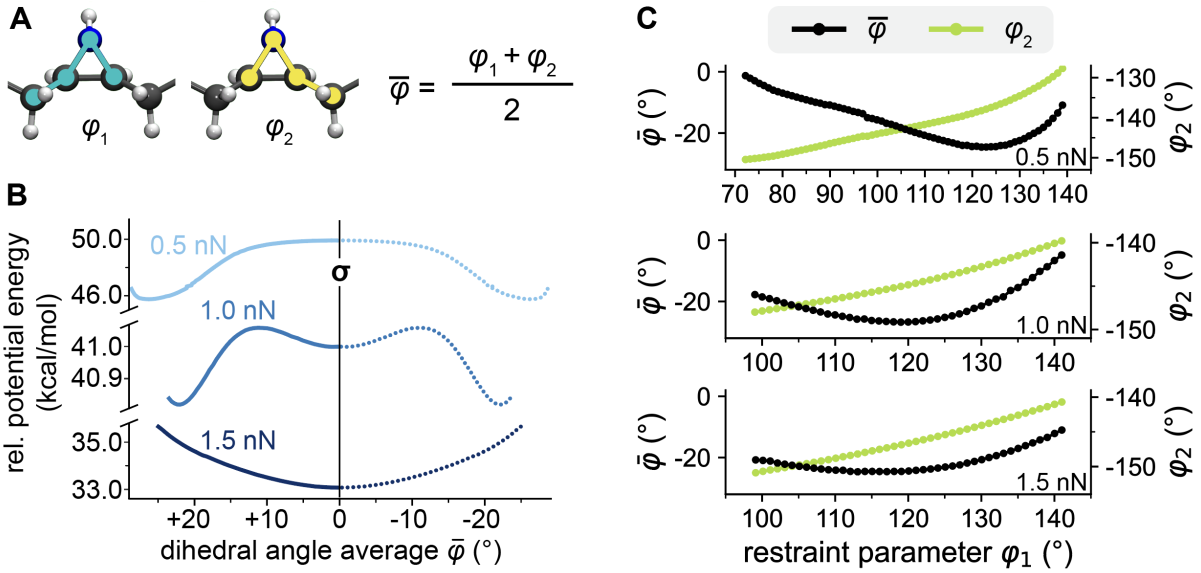

Figure S5: **A**: Definition of dihedral angles φ_1 and φ_2, and colletive variable $\bar{\varphi}$. **B**: Energetic profiles along the symmetry-breaking coordinate at three different forces. Profiles were computed for positive values of $\bar{\varphi}$ (solid lines), then mirrored to obtain the full range (dotted lines). **C**: Changes in φ_2 and $\bar{\varphi}$ during the restraint scans.

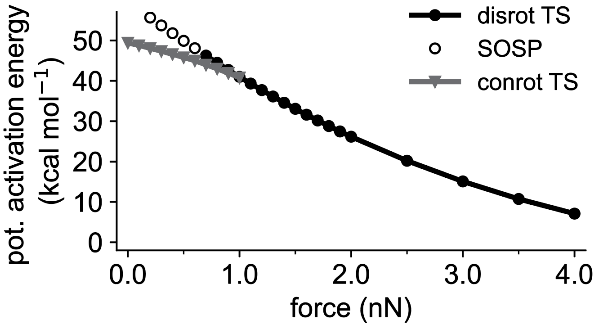

Figure S6: Force-modified activation energies (B3LYP/6-31G*) of the conrotatory and disrotatory ring-opening reactions of *cis*-dimethyl aziridine.

Table S1: Force-modified potential activation energies for the con- and disrotatory ring-opening of *cis*-dimethyl aziridine. All values listed in kcal/mol, dashes denote forces where no transition structure exists for the given reaction. Italics and brackets mark energies of second order saddle points instead of true transition structures, bold text marks the transition state rupture force

force (nN)	conrotatory ring-opening	disrotatory ring-opening
0.0	49.4	–
0.1	48.8	–
0.2	48.1	(*55.7*)
0.3	47.3	(*53.7*)
0.4	46.6	(*51.8*)
0.5	45.8	(*49.9*)
0.6	45.0	(*48.1*)
0.7	44.0	46.3
0.8	43.0	44.5
0.9	41.9	42.7
**1.0**	40.8	41.0
1.1	–	39.3
1.2	–	37.7
1.3	–	36.1
1.4	–	34.6
1.5	–	33.1
1.6	–	31.6
1.7	–	30.2
1.8	–	28.8
1.9	–	27.5
2.0	–	26.2
2.5	–	20.2
3.0	–	15.1
3.5	–	10.7
4.0	–	7.1

S.4 Interpolated potential energy surface

To better visualize the second order saddle point, an approximate potential energy surface was constructed by computing single-point energies at the B3LYP+D3/6-31G* level on interpolated geometries between the conrotatory and disrotatory IRCs of *cis*-dipropyl aziridine at 0.7 nN. This is shown in the following Figure S7. The two energy-lowering motions at this point are the symmetric, disrotatory ring-opening motion (Mode A, orange) and the asymmetric twisting mode connecting the two conrotatory transition structures (Mode B, purple).

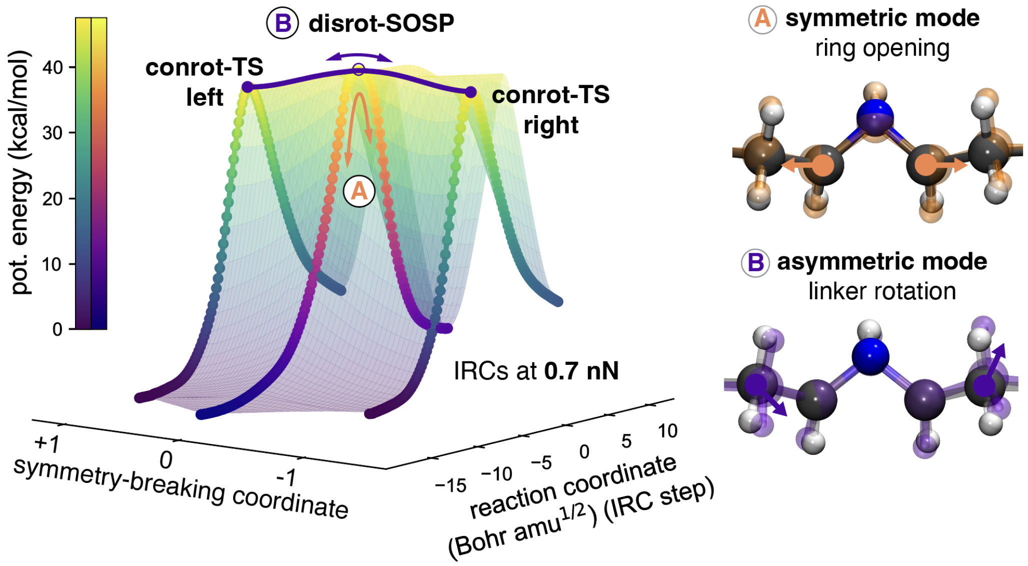

Figure S7: Force-modified potential energy surface (0.7 nN external force) obtained from single-point energies on geometries interpolated between the conrotatory and disrotatory IRCs.

S.5 Vibrational analysis along the disrotatory IRCs of *cis*-dipropyl aziridine

The topology of the potential energy surface of *cis*-dipropyl aziridine was investigated through vibrational analysis, which was performed along the disrotatory reaction path's IRC at various forces. From this vibrational analysis, information on the curvature of the potential energy surface can be gained: In the eigenvalue spectrum of the Hessian matrix, positive eigenvalues indicate an upward curvature along the corresponding vibrational mode. This means that deflection along this mode would lead to an increase in energy of the system. Negative eigenvalues denote a downward curvature along a specific mode, meaning that this molecular motion is an energy lowering motion for the system, i.e. a pathway leading to a more favorable structure. The frequencies of the two modes presented in Figure S7 were tracked along the IRC at different forces. At 1.0 nN and 1.1 nN, the stationary structure (reaction coordinate of 0.0 Bohr amu$^{1/2}$) is a SOSP, as both modes have a downward curvature. At external forces of 1.2 nN and up, the curvature of the second mode B is positive, showing the stationary point to be a true transition structure with only one concave-down energy-lowering mode.

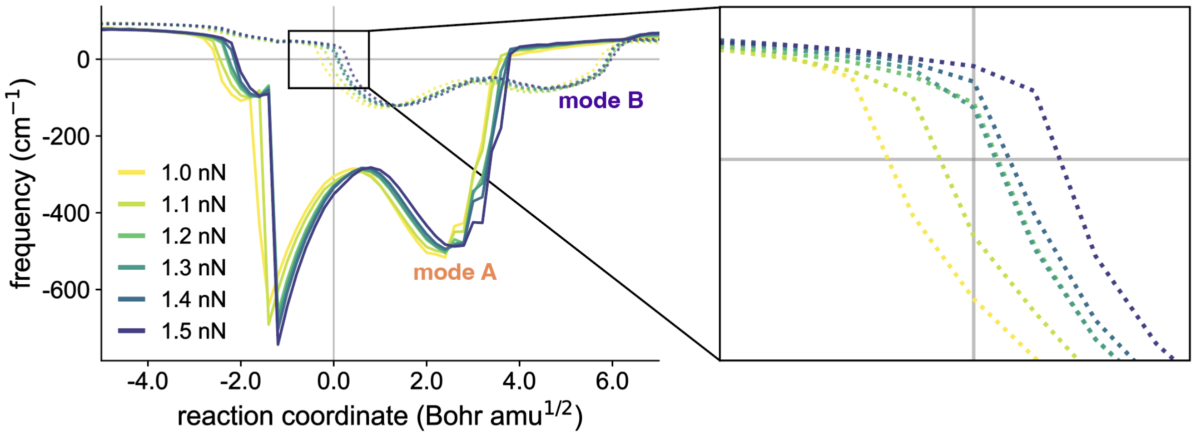

Figure S8: Vibrational analysis along the disrotatory reaction path of *cis*-dipropyl aziridine.

S.6 Diradical character of the studied mechanophores

The diradical character along the IRC of the thermally favored conrotatory reaction without external force was computed to serve as a predictor for F_{TSR}. *Cis*-dipropyl substituted cyclobutene (CBE), aziridine, and oxirane were studied. For each structure along the IRC, a CASSCF computation was made with the active orbitals relevant for the ring-opening reaction (see Figure S2).[41] From the occupation numbers n_i of the natural orbitals, the *Effective Number of Unpaired Electrons* (ENUE) can be computed at every IRC step according to[42]

$$\mathrm{ENUE} = \sum_i n_i^2 \cdot (n_i - 2)^2, \quad (1)$$

and regarded as a measure of diradical character: an ENUE of 2.0 represents a pure diradical structure, while an ENUE of 0.0 results from a closed-shell structure. In-between values can be understood as mixed electronic states with partial diradical character. By considering an ideal diradical to have an ENUE of 2.0, the percentage of diradical character for mixed species can be estimated from their ENUE *via* linear interpolation.

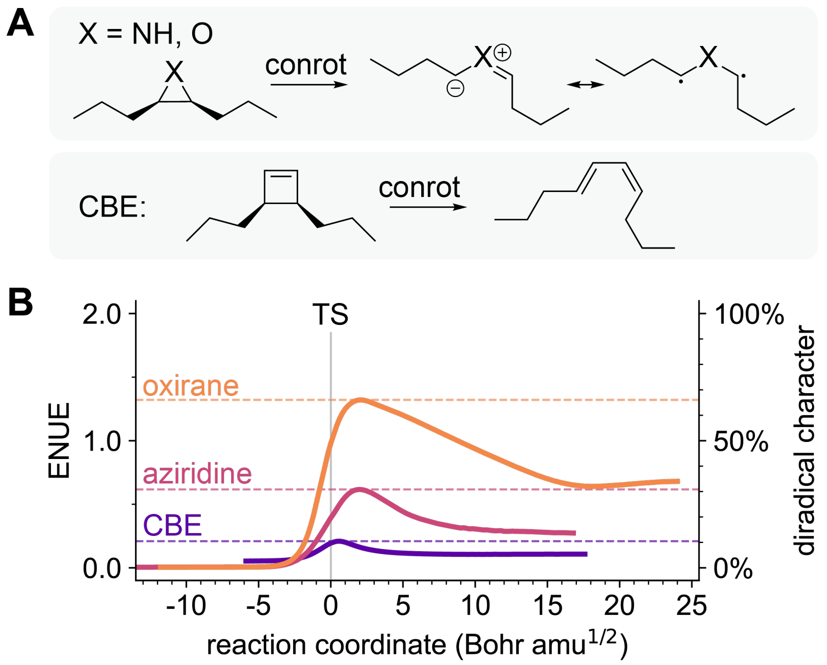

Figure S9: **A**: Conrotatory ring-opening reactions of *cis*-dipropyl oxirane, aziridine, and cyclobutene (CBE). **B**: Effective Number of Unpaired Electrons (ENUE) and derived percentage of diradical character along the IRC of the conrotatory ring-opening reactions of oxirane (orange), aziridine (red), and CBE (purple) without external force. The vertical grey line marks the transition structure along the reaction path.

Figure S9 shows the ENUE along the force-free conrotatory reaction path for each of the three mechanophores, alongside the estimated diradical character. For the cyclobutene mechanophore, the ENUE barely deviates from 0, reaching a maximum of only 0.21. This confirms the known closed-shell character of the WH-favored reaction.[43,44] In the diene product the ENUE again drops close to 0, in agreement with the expected electronic nature. The slight deviations from 0 in both reactant and product structure result from the small energetic gap between the bonding and antibonding π-orbitals, which leads to a persistant partial occupation of the π^* LUMO. For aziridine and oxirane, the product of the ring-opening reaction is a ylidic structure, which can be understood as either a zwitterion or a diradical. This is reflected in the ENUE along these reaction pathways, which does not decrease to 0 in the product regions.[45,46] For aziridine, the ENUE reaches a maximum of 0.62 shortly past the transition structure, before decreasing to 0.27 in the product region. With a maximum diradical character of 31 % along the reaction pathway, and only around 14 % in the product, the ring-opening reaction of aziridine is dominated by the zwitterionic structure. The contributions of the diradical electronic state are greatest in the ring-opening reaction of oxirane. The ENUE maximum is 1.32, translating to a diradical character of 66 % despite the WH-favored nature of the conrotatory reaction. In the carbonyl ylide product the ENUE remains high at 0.68, making the diradical contributions comparably large at 34 %. For both aziridine and oxirane, the computed diradical character percentages are in qualitative agreement with values reported in literature for simplified structures.[45,46]

S.7 Linker effect on diradical character

We present the diradical character of the WH-allowed ring-opening reaction as one predictor for F_{TSR}, and the linker effect as another, separate modifier. One could hypothesize that the linker affects F_{TSR} by modifying the diradical character of the system. To disprove this, the ENUE along the 0.0 nN IRC of the conrotatory ring-opening reaction of *cis*-dipropyne cyclobutene was computed. F_{TSR} of this system is 0.84 nN. If indeed the linkers were modifying the diradical character of the reaction, the ENUE maximum of this reaction should be similar in magnitude to that computed for *cis*-dipropyl oxirane, which has an F_{TSR} of 0.66 nN.

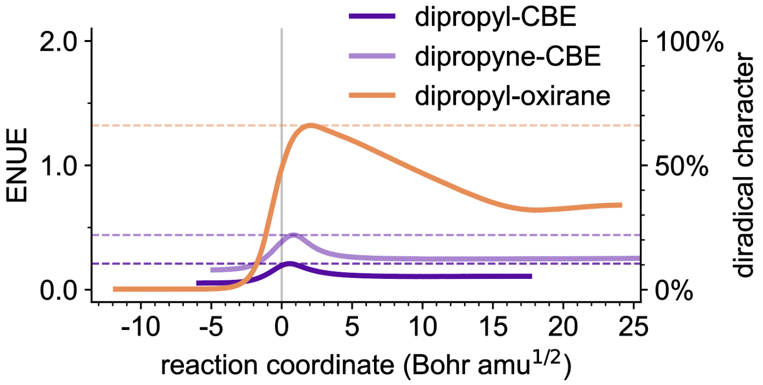

Figure S10: Effective Number of Unpaired Electrons (ENUE) and derived percentage of diradical character along the IRC of the conrotatory ring-opening reactions of dipropyl-oxirane (yellow), dipropyne-CBE (light purple), and dipropyl-CBE (dark purple) without external force.

The comparison of ENUEs in Figure S10 shows that this is not the case. For dipropyne cyclobutene, the ENUE maximum is only marginally higher than for the dipropyl-substituted mechanophore. Overall, a slight upwards shift of the ENUE along the entire reaction path can be observed, which is caused by the larger active space necessary for the CASSCF computation of the dipropyne linker.

S.8 Spin density and ENUE along the reaction pathways of conrotatory and disrotatory ring-opening reactions

In addition to the ENUE along the force-free conrotatory IRCs, the spin densities obtained from broken-symmetry DFT computations can be compared. In Figure S11 they are shown alongside the ENUE. Additionally, spin densities and ENUE along the disrotatory ring-opening reaction path at 2.0 nN are depicted, from which the pronounced diradical character of this reaction is evident for all three mechanophores.

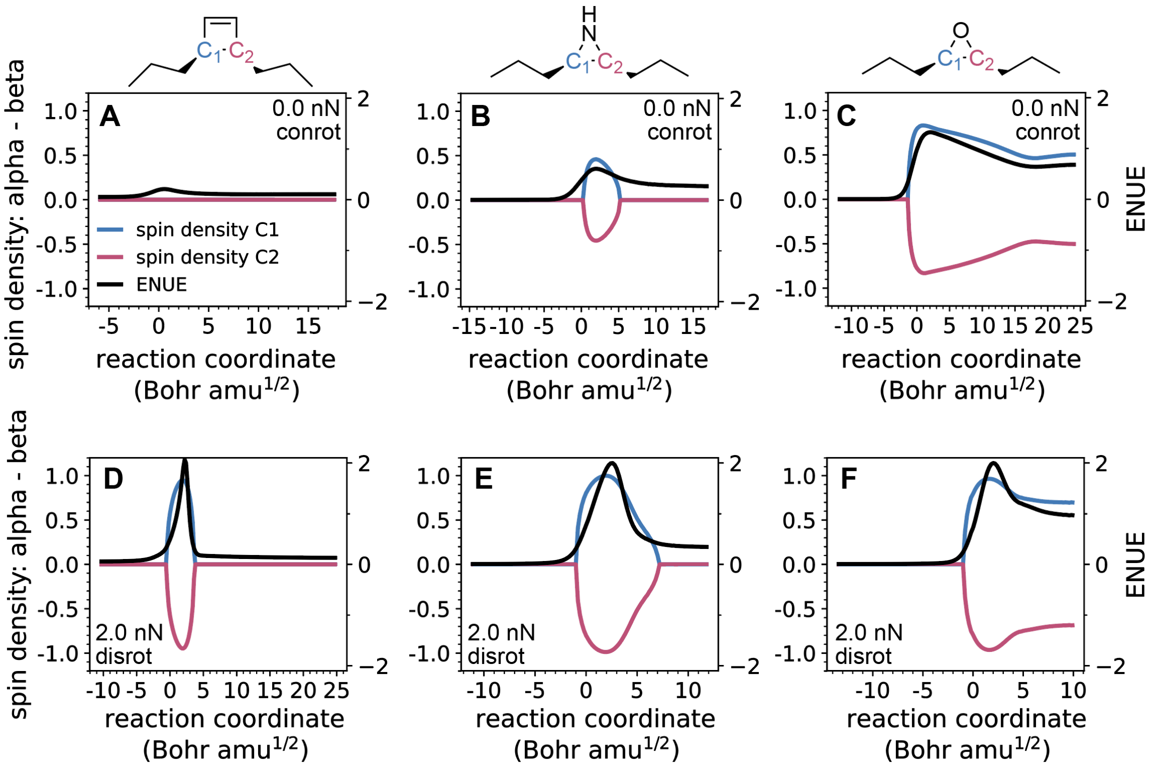

Figure S11: Broken symmetry DFT spin density (alpha - beta) at the two carbon atoms of the scissile bond and ENUE along the IRC of the conrotatory (**A**-**C**) and disrotatory (**D**-**F**) ring-opening reaction **A**/**D**: *cis*-dipropyl-cyclobutene, **B**/**E**: *cis*-dipropyl-aziridine, and **C**/**F**: *cis*-dipropyl-oxirane.

S.9 Comparison between *cis*-diphenyl- and *cis*-dipropyne aziridine

From experiments, phenyl linkers are known to strongly activate mechanochemical reactions.[47] The Restoring Force Triangle (RTF) predicts that phenyl linkers have similar properties to propyne linkers, which we have chosen for our study of transition state rupture events to avoid the complications arising from conformers inherent in phenyl-substituted systems. To prove that the two linkers behave similarly within the scope of our study, force-modified activation energies and F_{TSR} were computed at the CASPT2/cc-pVTZ // B3LYP/6-31G* level of theory for *cis*-diphenyl aziridine, and compared to *cis*-dipropyne aziridine.

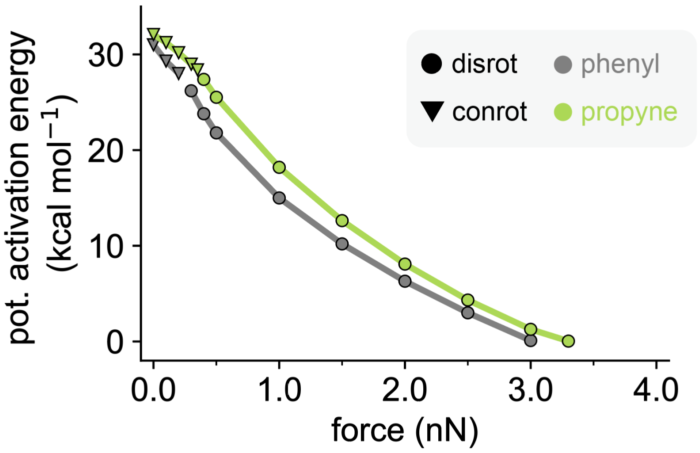

Figure S12: Force-modified activation energies of the conrotatory and disrotatory ring-opening reactions of *cis*-diphenyl aziridine (grey) and *cis*-dipropyne aziridine (green).

Table S2: Force-modified potential activation energies for the con- and disrotatory ring-opening of *cis*-diphenyl aziridine, computed at the CASPT2/cc-pVTZ // B3LYP/6-31G* level of theory. All values listed in kcal/mol, dashes denote forces where no transition structure exists for the given reaction. Bold text marks the transition state rupture force

force (nN)	conrotatory ring-opening	disrotatory ring-opening
0.0	31.0	–
0.1	29.3	–
**0.2**	28.0	–
0.3	–	26.2
0.4	–	23.8
0.5	–	21.8
1.0	–	15.0
1.5	–	10.2
2.0	–	6.3
2.5	–	3.0
3.0	–	0.1

S.10 AISMD simulations of the phthalimide migration reaction

Ab initio steered molecular dynamics (AISMD) calculations for the investigation of the *N*-phthalimido aziridine's post-activation reactivity were performed in the TeraChem program suite with GPU acceleration.[5–7] A constant force was applied to the two steered carbon atoms, which are the same terminal carbon atoms as in the optimization of stationary points. All AISMD simulations were performed using the B3LYP+D3/6-31G* level of theory, in an NVE-ensemble with a step size of 1 fs. The same implicit solvation model as for the optimization of stationary points was used in the simulations. Trajectories

were initialized from both the conrotatory and disrotatory transition states of the *N*-phthalimido aziridine compound, at different forces: 1.0 nN and 1.5 nN for the conrotatory reaction, 1.5 nN and 2.0 nN for the disrotatory reaction. The external force was assumed to be constant on the picosecond timescale of the AISMD simulations. Harmonic Wigner sampling was used for sampling of the initial conditions (structures and initial momenta) for all non-imaginary modes at the corresponding FM-PES transition state structures at 300 K. Position displacements were made along the imaginary mode, ensuring that all sampled initial conditions are on the same side of the transition state with a velocity that points away from the transition state towards the product minimum. 20 trajectories were run per force, each for a total simulation time of 10 ps.

The exothermicity of the dissociation reaction, in which the N–N bond between azomethine ylide and phthalimide is cleaved, causes the two resulting fragments to drift apart rapidly in some trajectories (see Figure S13 B). This is unphysical, as it neglects the caging effect of surrounding solvent molecules, which would prevent this in a real system. As the simulation of this cage effect requires a large number of solvent molecules, we instead opted to use spherical boundary conditions (SBC) as a means to prevent excessive diffusion at a reasonable computational cost.[48,49] We used the SBC implementation in TeraChem, with a radius of 10 Å and a force constant of 3.0 kcal/(mol·Å^2).

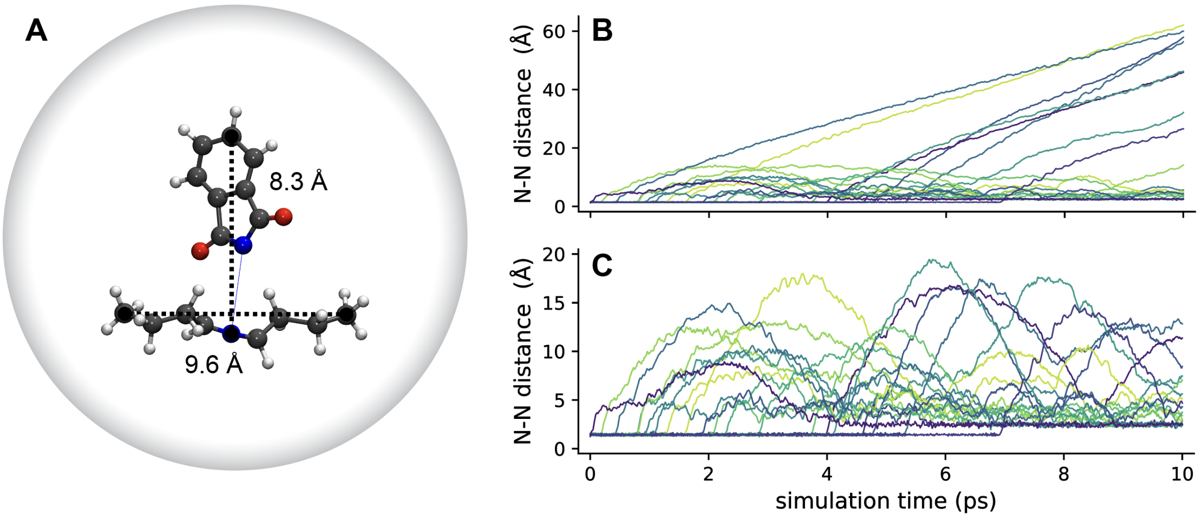

Figure S13: **A**: Setup of the spherical boundary conditions (SBC) used in the AISMD simulations, with a sphere radius of 10 Å. **B**: N–N distance during AISMD simulations started from the disrotatory transition state at 1.5 nN without SBC. Colors were chosen to visually distinguish the trajectories. **C**: N–N distance during AISMD simulations started from the disrotatory transition state at 1.5 nN with SBC. Colors were chosen to visually distinguish the trajectories.

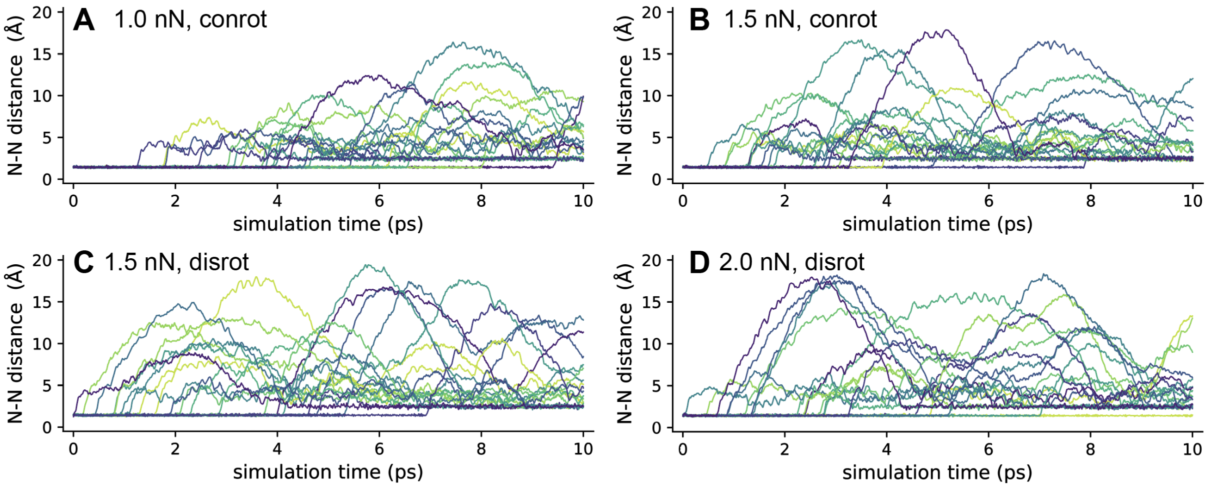

Figure S14: N–N distance during AISMD simulations with spherical boundary conditions. Colors were chosen to visually distinguish the trajectories. **A**: Started from the conrotatory transition state at 1.0 nN external force. **B**: Started from the conrotatory transition state at 1.5 nN external force. **C**: Started from the disrotatory transition state at 1.5 nN external force. **D**: Started from the disrotatory transition state at 2.0 nN external force.

Figure S14 shows that there is very little difference in system behavior during the four different simulated scenarios. The only notable observation is the slightly delayed onset of follow-up reactions at 1.0 nN. Two side reactions were observed during the simulation: deprotonation of the cationic fragment by the phthalimide anion, and formation of a carboximidate through nucleophilic attack of the phthalimide oxygen. These reactions will be discussed in the next section.

Table S3: Results of the AISMD simulations with spherical boundary conditions

			main reaction			side products	
	force	no. of runs	ylide	intermediate fragments	imine	deprotonation	carboximidate
conrot	1.0 nN	20	–	12 (60%)	6 (30%)	1 (5%)	1 (5%)
	1.5 nN	20	–	3 (15%)	11 (55%)	4 (20%)	2 (10%)
disrot	1.5 nN	20	–	9 (45%)	7 (35%)	2 (10%)	2 (10%)
	2.0 nN	20	1 (5%)	10 (50%)	7 (35%)	2 (10%)	–
total		80	1 (1.3%)	34 (42.5%)	31 (38.8%)	9 (11.3)	5 (6.3%)

Representative trajectories are included as movies:

Movie S1: Formation of the imine product, started from the disrotatory TS at 1.5 nN ext. force

Movie S2: Deprotonation by the phthalimide anion, started from the disrotatory TS at 1.5 nN ext. force

Movie S3: Formation of the carboximidate side product, started from the disrotatory TS at 1.5 nN ext. force

S.11 Side reactions found in AISMD simulations

The deprotonation reaction was observed both in α and in β position during the AISMD simulations. The product of the former is an unstable zwitterionic structure, while the latter reaction forms a stable alkene. Figure S15 shows the potential energy profile of both reactions relative to the energy of the phthalimide/azaallenium intermediate.

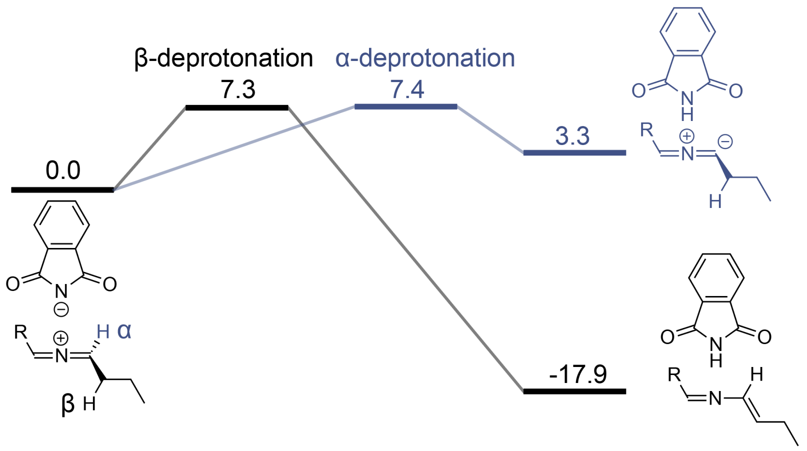

Figure S15: Potential energy profile computed at the B3LYP+D3/6-31G* level of theory without external force for deprotonation reactions in α- (blue) and β-position (black) observed in the AISMD trajectories.

The other side reaction that occurs in the AISMD simulations is a nucleophilic attack of the phthalimide oxygen at one of the carbon atoms neighboring the azaallenium nitrogen, resulting in a carboximidate. The barrier for the backwards reaction is relatively low, and in some trajectories the formation and subsequent reverse reaction back to the fragmented intermediate was observed. In Table S3, only those trajectories where the carboximidate structure is present at the end of the simulation were counted.

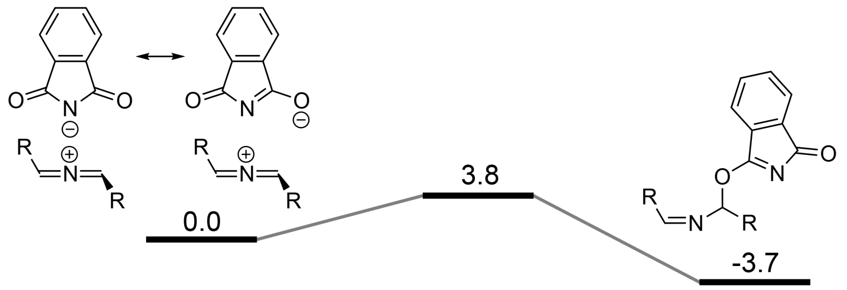

Figure S16: Potential energy profile computed at the B3LYP+D3/6-31G* level of theory without external force for carboximidate formation observed in the AISMD trajectories.

S.12 Effect of external force on *N*-phthalimido azomethine ylide follow-up reactions

In the main text, the potential energies of the *N*-phthalimido azomethine ylide follow-up reactions are reported without external force, at the CASPT2/cc-pVTZ // B3LYP+D3/6-31G* level of theory. The here presented force-modified energies were computed at the B3LYP+D3/6-31G* level, without the additional CASPT2 energy correction. For the recombination reaction that occurs as the final step of the ylide-to-imine transformation the transition structure could only be optimized without external force, hence no force dependence is reported here. The bimolecular intermediate could only be optimized up to forces of 2.4 nN, at greater forces the optimization converged to the imine product instead.

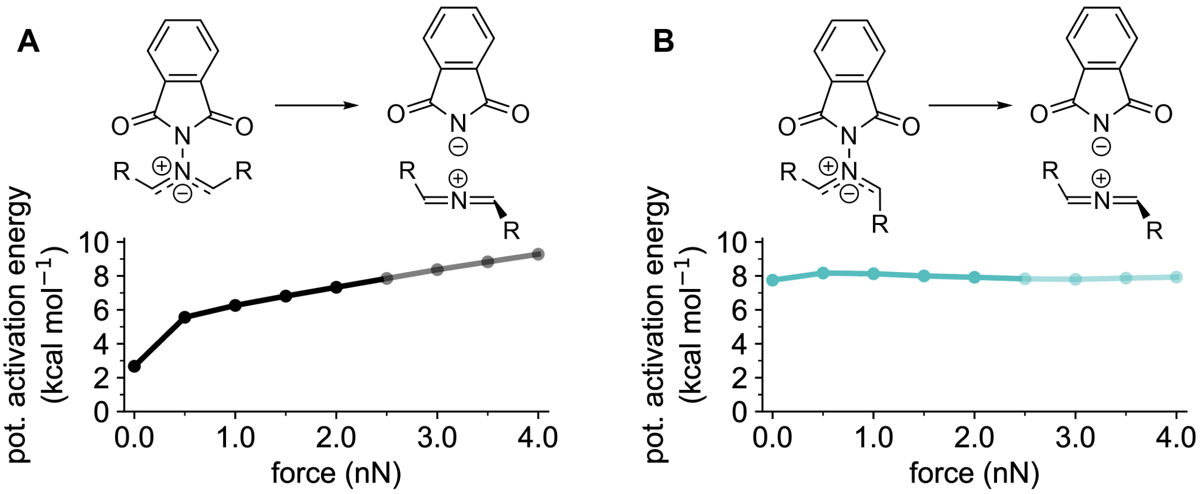

Figure S17: Force-modified potential activation energies of the N–N bond dissociaton reactions of the *N*-phthalimido ylides. **A**: W-ylide **B**: S-ylide. Transparent icons mark the force range in which the ionic intermediate could not be optimized and the reaction proceeds to the imine directly.

Table S4: Force-modified potential activation energy for the N–N bond dissociaton reactions of the W- and S-ylide generated from the *cis*-dipropyl-*N*-phthalimido aziridine ring-opening reactions, computed at the B3LYP/6-31G* level of theory. All values are listed in kcal/mol

force (nN)	W-ylide dissociation reaction	S-ylide dissociation reaction
0.0	2.7	7.8
0.5	5.6	8.2
1.0	6.3	8.1
1.5	6.8	8.0
2.0	7.3	8.0
2.5	7.9	7.9
3.0	8.4	7.8
3.5	8.8	7.9
4.0	9.3	8.0

S.13 Isomerization reaction of the imine product

The imine product of the phthalimide's migration reaction can exist in either *E* or *Z* configuration. Both isomers are generated in the recombination reaction between azaallenium cation and phthalimide anion, as observed in the AISMD trajectories. Conversion between the two isomers is possible *via* an isomerization reaction, which favors the more stable *E*-imine, especially under external force.

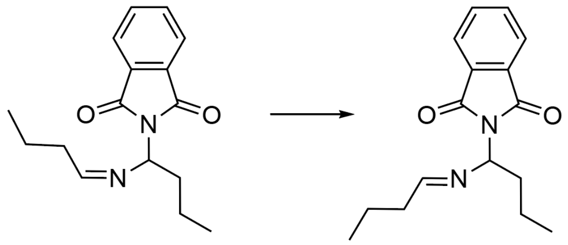

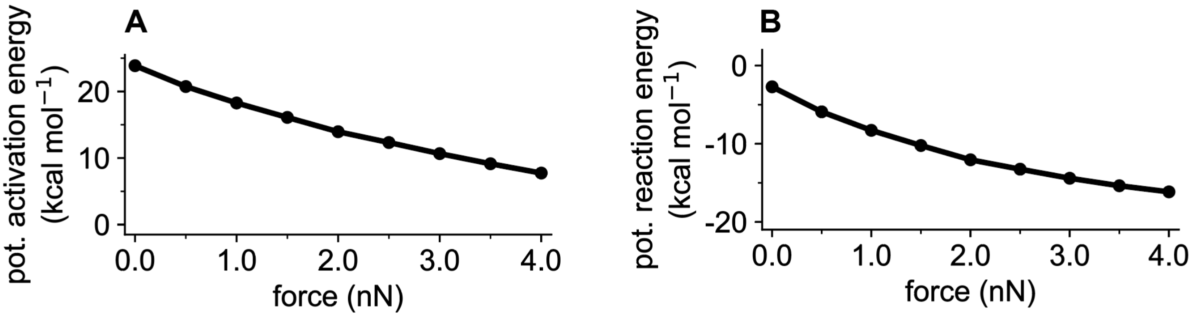

Figure S18: **A**: Force-modified potential activation energy and **B**: force-modified potential reaction energy of the *Z* to *E* isomerization reaction of the imine resulting from the phthalimide migration reaction.

Table S5: Force-modified potential activation energy and reaction energy for the isomerization from *Z*- to *E*-imine, computed at the B3LYP/6-31G* level of theory

force (nN)	pot. activation energy (kcal/mol)	pot. reaction energy (kcal/mol)
0.0	23.9	-2.7
0.5	20.7	-5.9
1.0	18.3	-8.3
1.5	16.1	-10.2
2.0	14.0	-12.1
2.5	12.3	-13.3
3.0	10.7	-14.4
3.5	9.1	-15.4
4.0	7.7	-16.2

S.14 Ring-opening reactions of *trans*-substituted experimentally studied aziridines

For *trans*-substituted 4 π-electron mechanophores, the WH-allowed conrotatory reaction is also the force-assisted reaction motion.[50] Therefore the disrotatory reaction does not proceed, and the conrotatory ring-opening has no competition. The product of the conrotatory ring-opening reaction of *trans*-substituted aziridines is a W-shaped azomethine ylide. This is consistent with the observation made by Jung and Yoon: When *trans*-diester-*N*-methoxybenzene aziridine was sonicated in the presence of dimethyl acetylenedicarboxylate (DMAD), a *cis*-configured product was obtained, as expected from the reaction of between a W-ylide and a dipolarophile.[51,52]

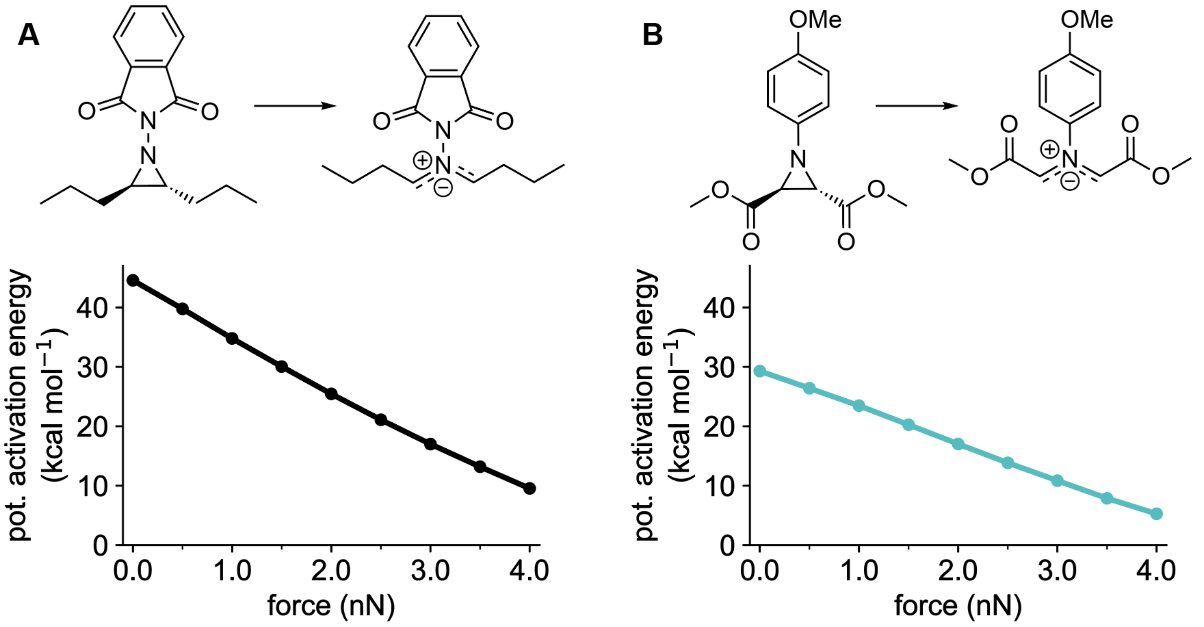

Figure S19: Force-modified potential activation energies of the conrotatory ring-opening reaction for **A**: *trans*-dipropyl-*N*-phthalimido aziridine and **B**: *trans*-diester-*N*-methoxybenzene aziridine.

Table S6: Force-modified potential activation energy for the conrotatory ring-opening of *trans*-dipropyl-substituted *N*-phthalimido aziridine and *trans*-diester-substituted *N*-methoxybenzene aziridine, computed at the B3LYP/6-31G* level of theory. All values are listed in kcal/mol

force (nN)	*cis*-dipropyl-*N*-phthalimido aziridine	*cis*-diester-*N*-methoxybenzene aziridine
0.0	44.6	29.3
0.5	39.8	26.4
1.0	34.8	23.5
1.5	30.0	20.3
2.0	25.4	17.0
2.5	21.1	13.8
3.0	17.0	10.8
3.5	13.2	7.9
4.0	9.5	5.3

S.15 Ylide isomerization reactions in experimentally studied aziridines

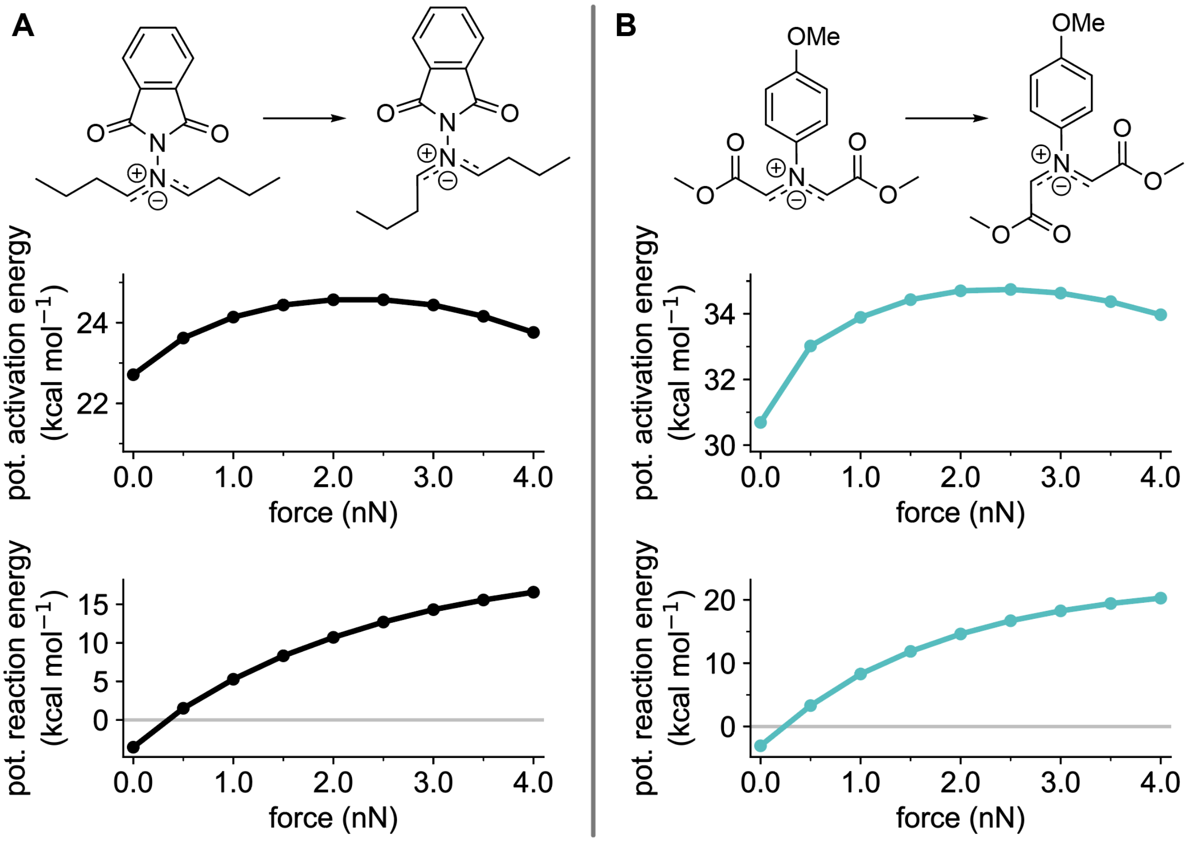

Figure S20: Force-modified potential activation energies (top plot) and reaction energies (bottom plot) of the W- to S-ylide isomerization reaction for **A**: dipropyl-*N*-phthalimido ylide and **B**: diester-*N*-methoxybenzene ylide.

Table S7: Force-modified potential activation energy and reaction energy for the isomerization from W- to S-ylide of dipropyl-substituted *N*-phthalimido aziridine, computed at the B3LYP/6-31G* level of theory

force (nN)	pot. activation energy (kcal/mol)	pot. reaction energy (kcal/mol)
0.0	22.7	-3.5
0.5	23.6	1.5
1.0	24.1	5.3
1.5	24.4	8.3
2.0	24.6	10.7
2.5	24.6	12.7
3.0	24.4	14.3
3.5	24.2	15.6
4.0	23.8	16.6

Table S8: Force-modified potential activation energy and reaction energy for the isomerization from W- to S-ylide of diester-substituted *N*-methoxybenzene aziridine, computed at the B3LYP/6-31G* level of theory

force (nN)	pot. activation energy (kcal/mol)	pot. reaction energy (kcal/mol)
0.0	30.7	-3.0
0.5	33.0	3.3
1.0	33.9	8.3
1.5	34.4	11.9
2.0	34.7	14.6
2.5	34.7	16.7
3.0	34.6	18.3
3.5	34.4	19.4
4.0	34.0	20.3

S.16 Force-modified activation energies presented in the main text figures

All energies listed here are reported in the main text figures, and were computed at the CASPT2/cc-pVTZ // B3LYP+D3/6-31G* level of theory.

Table S9: Force-modified potential activation energies for the con- and disrotatory ring-opening of *cis*-dipropyl cyclobutene. All values listed in kcal/mol, dashes denote forces where no transition structure exists for the given reaction. Italics and brackets mark energies of second order saddle points instead of true transition structures

force (nN)	conrotatory ring-opening	disrotatory ring-opening
0.0	32.9	–
0.1	33.1	–
0.2	32.6	–
0.3	32.0	–
0.4	31.2	(*41.5*)
0.5	30.4	(*38.9*)
0.6	29.4	(*36.3*)
0.7	28.5	(*34.1*)
0.8	27.5	(*31.7*)
0.9	26.5	(*29.7*)
1.0	25.4	(*27.7*)
1.1	24.5	(*25.8*)
1.2	23.3	(*23.9*)
1.3	22.4	22.2
1.4	21.2	20.6
1.5	20.1	19.0
1.6	18.9	17.5
1.7	17.8	16.1
1.8	16.6	14.6
1.9	15.4	13.4
2.0	14.4	12.1
2.1	13.0	10.8
2.2	11.8	9.7
2.3	10.5	8.6
2.4	9.2	7.6
2.5	7.9	6.5
2.6	6.6	5.4
2.7	5.2	4.6
2.8	3.9	3.6
2.9	2.5	2.8
3.0	1.1	1.8
3.07	0.1	

Table S10: Force-modified potential activation energies for the con- and disrotatory ring-opening of *cis*-diester cyclobutene. All values listed in kcal/mol, dashes denote forces where no transition structure exists for the given reaction

force (nN)	conrotatory ring-opening	disrotatory ring-opening
0.0	27.8	–
0.1	28.7	–
0.2	29.4	–
0.3	29.8	–
0.4	30.0	–
0.5	29.9	–
0.6	29.7	–
0.7	29.4	–
0.8	29.0	–
0.9	28.3	–
1.0	27.7	–
1.1	27.1	–
1.2	26.4	–
1.3	25.6	–
1.4	24.7	–
1.5	23.8	–
1.6	22.7	–
1.7	21.7	–
1.8	20.7	–
1.9	19.8	–
2.0	18.7	–
2.1	17.6	–
2.2	16.7	14.4
2.3	15.4	13.5
2.4	14.4	12.4
2.5	12.8	11.3
2.6	11.8	10.2
2.7	10.6	9.2
2.8	9.4	8.1
2.9	8.2	7.1
3.0	7.0	6.2
3.1	5.7	5.2
3.2	4.5	4.3
3.3	3.1	3.4
3.4	1.7	2.4
3.5	0.5	1.6
3.51	0.4	

Table S11: Force-modified potential activation energies for the con- and disrotatory ring-opening of *cis*-dipropyne cyclobutene. All values listed in kcal/mol, dashes denote forces where no transition structure exists for the given reaction. Bold text marks the transition state rupture force

force (nN)	conrotatory ring-opening	disrotatory ring-opening
0.0	25.1	–
0.1	25.8	–
0.2	25.6	28.6
0.3	24.9	26.4
0.4	23.9	24.2
0.5	22.7	22.2
0.6	21.4	20.3
0.7	19.9	18.5
0.8	18.2	16.7
**0.84**	17.6	
0.9	–	15.1
1.0	–	13.6
1.5	–	7.0
2.0	–	2.3
2.5	–	0.1

Table S12: Force-modified potential activation energies for the con- and disrotatory ring-opening of *cis*-dipropyl aziridine. All values listed in kcal/mol, dashes denote forces where no transition structure exists for the given reaction. Italics and brackets mark energies of second order saddle points instead of true transition structures, bold text marks the transition state rupture force

force (nN)	conrotatory ring-opening	disrotatory ring-opening
0.0	49.1	–
0.1	48.7	–
0.2	48.0	–
0.3	47.2	–
0.4	46.1	–
0.5	44.9	–
0.6	43.8	–
0.7	42.7	(*47.5*)
0.8	41.6	(*45.6*)
0.9	40.5	(*43.7*)
1.0	39.4	(*42.0*)
1.1	38.3	(*40.2*)
1.2	37.3	38.4
1.3	36.4	36.8
**1.4**	35.2	35.3
1.5	–	33.7
1.6	–	32.2
1.7	–	30.8
1.8	–	29.5
1.9	–	28.1
2.0	–	26.8
2.5	–	20.9
3.0	–	15.6
3.5	–	11.0
4.0	–	7.0

Table S13: Force-modified potential activation energies for the con- and disrotatory ring-opening of *cis*-diester aziridine. All values listed in kcal/mol, dashes denote forces where no transition structure exists for the given reaction. Italics and brackets mark energies of second order saddle points instead of true transition structures, bold text marks the transition state rupture force

force (nN)	conrotatory ring-opening	disrotatory ring-opening
0.0	31.4	–
0.5	31.9	–
1.0	29.4	–
1.4		(*27.0*)
1.5	26.1	(*25.9*)
1.6	25.4	(*24.9*)
1.7	24.6	(*23.8*)
1.8	23.7	22.8
1.9	22.5	21.7
2.0	21.4	20.8
2.1	20.3	19.8
**2.16**	19.6	
2.2	–	18.8
2.3	–	17.9
2.4	–	16.9
2.5	–	16.0
3.0	–	11.8
3.5	–	8.1
4.0	–	4.8

Table S14: Force-modified potential activation energies for the con- and disrotatory ring-opening of *cis*-dipropyne aziridine. All values listed in kcal/mol, dashes denote forces where no transition structure exists for the given reaction. Bold text marks the transition state rupture force

force (nN)	conrotatory ring-opening	disrotatory ring-opening
0.0	32.1	–
0.1	31.2	–
0.2	30.2	–
0.3	29.0	–
**0.35**	28.4	
0.4	–	27.4
0.5	–	25.5
1.0	–	18.2
1.5	–	12.6
2.0	–	8.1
2.5	–	4.3
3.0	–	1.3
3.3	–	0.0

Table S15: Force-modified potential activation energies for the con- and disrotatory ring-opening of *cis*-dipropyl oxirane. All values listed in kcal/mol, dashes denote forces where no transition structure exists for the given reaction. Bold text marks the transition state rupture force

force (nN)	conrotatory ring-opening	disrotatory ring-opening
0.0	56.6	–
0.1	56.4	–
0.2	55.6	–
0.3	54.7	–
0.4	53.8	–
0.5	52.8	–
0.6	51.8	–
**0.66**	51.3	
0.7	–	52.0
0.8	–	50.2
0.9	–	48.5
1.0	–	46.9
1.1	–	45.3
1.2	–	43.8
1.3	–	42.3
1.4	–	40.9
1.5	–	39.8
2.0	–	33.3
2.5	–	27.7
3.0	–	22.6
3.5	–	18.1
4.0	–	13.9

Table S16: Force-modified potential activation energies for the con- and disrotatory ring-opening of *cis*-diester oxirane. All values listed in kcal/mol, dashes denote forces where no transition structure exists for the given reaction. Italics and brackets mark energies of second order saddle points instead of true transition structures, bold text marks the transition state rupture force

force (nN)	conrotatory ring-opening	disrotatory ring-opening
0.0	41.4	–
0.1	41.3	–
0.2	40.1	–
0.3	38.9	–
0.4	38.2	–
0.5	37.6	–
0.6	37.0	–
0.7	36.4	(*38.9*)
0.8	36.0	(*37.9*)
0.9	35.6	36.9
1.0	35.2	35.8
**1.04**	34.9	
1.5	–	30.4
2.0	–	25.5
2.5	–	21.1
3.0	–	17.0
3.5	–	13.3
4.0	–	10.0

Table S17: Force-modified potential activation energies for the con- and disrotatory ring-opening of *cis*-dipropyne oxirane. All values listed in kcal/mol, dashes denote forces where no transition structure exists for the given reaction. Bold text marks the transition state rupture force

force (nN)	conrotatory ring-opening	disrotatory ring-opening
0.0	38.2	–
0.1	37.7	–
0.2	37.4	38.2
**0.21**	37.3	
0.3	–	36.2
0.4	–	34.4
0.5	–	32.8
0.6	–	31.2
0.7	–	29.8
0.8	–	28.4
0.9	–	27.0
1.0	–	25.8
1.5	–	20.2
2.0	–	15.4
2.5	–	11.3
3.0	–	7.8
3.5	–	4.8
4.0	–	2.3

Table S18: Force-modified potential activation energies for the con- and disrotatory ring-opening of *cis*-dipropyl-*N*-methoxybenzene aziridine. All values listed in kcal/mol, dashes denote forces where no transition structure exists for the given reaction. Bold text marks the transition state rupture force

force (nN)	conrotatory ring-opening	disrotatory ring-opening
0.0	44.5	–
0.5	41.4	–
1.0	36.8	–
1.5	31.3	–
1.6	30.2	–
1.7	29.0	–
1.8	27.8	–
1.9	26.4	–
**2.0**	25.5	–
2.1	–	24.3
2.2	–	22.9
2.3	–	21.7
2.4	–	20.4
2.5	–	19.2
3.0	–	13.6
3.5	–	8.9
4.0	–	4.8

Table S19: Force-modified potential activation energies for the con- and disrotatory ring-opening of *cis*-diester-*N*-methoxybenzene aziridine. All values listed in kcal/mol, dashes denote forces where no transition structure exists for the given reaction

force (nN)	conrotatory ring-opening	disrotatory ring-opening
0.0	30.1	–
0.5	28.0	–
1.0	26.1	–
1.5	23.2	–
2.0	19.6	–
2.5	15.8	–
3.0	11.8	–
3.1		–
3.2		8.5
3.3		7.8
3.4		7.1
3.5	7.9	6.4
3.6		5.7
3.7		5.0
3.8		4.4
3.9		3.7
4.0	4.2	3.2

Table S20: Force-modified potential activation energies for the con- and disrotatory ring-opening of *cis*-dipropyne-*N*-methoxybenzene aziridine. All values listed in kcal/mol, dashes denote forces where no transition structure exists for the given reaction. Bold text marks the transition state rupture force

force (nN)	conrotatory ring-opening	disrotatory ring-opening
0.0	30.0	–
0.1	29.4	–
0.2	28.5	–
0.3	27.3	–
0.4	25.9	–
**0.5**	24.4	–
0.6	–	22.8
0.7	–	21.2
0.8	–	19.8
0.9	–	18.4
1.0	–	17.1
1.5	–	11.6
2.0	–	7.2
2.5	–	3.5
3.0	–	0.6

Table S21: Force-modified potential activation energies for the con- and disrotatory ring-opening of *cis*-dipropyl-*N*-phthalimido aziridine. All values listed in kcal/mol, dashes denote forces where no transition structure exists for the given reaction. Italics and brackets mark energies of second order saddle points instead of true transition structures, bold text marks the transition state rupture force

force (nN)	conrotatory ring-opening	disrotatory ring-opening
0.0	47.7	–
0.5	40.7	–
0.9	34.5	(*36.9*)
1.0	33.0	(*34.9*)
1.1	31.5	(*32.8*)
1.2	30.3	(*31.2*)
1.3	28.9	29.3
1.4	27.4	27.6
**1.5**	25.8	26.0
1.6	–	
1.7	–	
1.8	–	
1.9	–	
2.0	–	18.1
2.5	–	12.9
3.0	–	8.0
3.5	–	3.8

References

[1] J. Kästner, J. M. Carr, T. W. Keal, W. Thiel, A. Wander, and P. Sherwood, *J. Phys. Chem. A*, Vol. 113, Nr. 43, S. 11 856–11 865, 2009.

[2] D. C. Liu and J. Nocedal, *Math. Program.*, Vol. 45, Nr. 1-3, S. 503–528, 1989.

[3] G. Henkelman and H. Jónsson, *J. Chem. Phys.*, Vol. 111, Nr. 15, S. 7010–7022, 1999.

[4] J. Kästner and P. Sherwood, *Chem. Phys.*, Vol. 128, Nr. 1, S. 014106, 2008.

[5] I. S. Ufimtsev and T. J. Martinez, *J. Chem. Theory Comput.*, Vol. 5, Nr. 10, S. 2619–2628, 2009.

[6] S. Seritan *et al.*, *Wiley Interdiscip. Rev. Comput. Mol. Sci.*, Vol. 11, Nr. 2, S. e1494, 2021.

[7] I. S. Ufimtsev and T. J. Martínez, *J. Chem. Theory Comput.*, Vol. 4, Nr. 2, S. 222–231, 2008.

[8] H.-J. Werner, P. J. Knowles *et al.*, „MOLPRO, 2022.3 , a package of ab initio programs."

[9] H.-J. Werner, P. J. Knowles, G. Knizia, F. R. Manby, and M. Schütz, *Wiley Interdiscip. Rev. Comput. Mol. Sci.*, Vol. 2, Nr. 2, S. 242–253, 2012.

[10] H.-J. Werner *et al.*, *J. Chem. Phys.*, Vol. 152, Nr. 14, S. 144107, 04 2020.

[11] S. Metz, J. Kästner, A. A. Sokol, T. W. Keal, and P. Sherwood, *Wiley Interdiscip. Rev. Comput. Mol. Sci.*, Vol. 4, Nr. 2, S. 101–110, 2014.

[12] M. T. Ong, J. Leiding, H. Tao, A. M. Virshup, and T. J. Martínez, *J. Am. Chem. Soc.*, Vol. 131, Nr. 18, S. 6377–6379, 2009.

[13] J. Ribas-Arino, M. Shiga, and D. Marx, *Angew. Chem., Int. Ed.*, Vol. 48, Nr. 23, S. 4190–4193, 2009.

[14] L. Noodleman, *J. Chem. Phys.*, Vol. 74, Nr. 10, S. 5737–5743, 1981.

[15] R. Ditchfield, W. J. Hehre, and J. A. Pople, *J. Chem. Phys.*, Vol. 54, S. 724–728, 1971.

[16] P. C. Hariharan and J. A. Pople, *Theor. Chim. Acta*, Vol. 28, S. 213–222, 1973.

[17] W. J. Hehre, R. Ditchfield, and J. A. Pople, *J. Chem. Phys.*, Vol. 56, S. 2257–2261, 1972.

[18] A. D. Becke, *Chem. Phys.*, Vol. 98, Nr. 492, S. 5648–5652, 1993.

[19] C. Lee, W. Yang, and R. Parr, *Phys. Rev. B*, Vol. 37, Nr. 2, S. 785–789, 1988.

[20] S. H. Vosko, L. Wilk, and M. Nusair, *Can. J. Phys.*, Vol. 58, Nr. 8, S. 1200–1211, 1980.

[21] P. S. F. D. C. Chabalowski and M. Frisch, *J. Phys. Chem*, Vol. 98, S. 11 623–11 627, 1994.

[22] S. Grimme, J. Antony, S. Ehrlich, and H. Krieg, *J. Chem. Phys.*, Vol. 132, Nr. 15, S. 154104, 04 2010.

[23] P. Pulay, *J. Comput. Chem.*, Vol. 3, Nr. 4, S. 556–560, 1982.

[24] K. Andersson, P.-Å. Malmqvist, and B. O. Roos, *J. Chem. Phys.*, Vol. 96, Nr. 2, S. 1218–1226, 1992.

[25] T. H. Dunning, *J. Chem. Phys.*, Vol. 90, S. 1007–1023, 1989.

[26] Y. Liu *et al.*, *Science*, Vol. 373, Nr. 6551, S. 208–212, 2021.

[27] S. Ding *et al.*, *J. Am. Chem. Soc.*, Vol. 146, Nr. 9, S. 6104–6113, 2024.

[28] A. Germann and J. Meisner, *J. Phys. Chem. A.*, Vol. 128, Nr. 47, S. 10 224–10 233, 2024.

[29] H.-J. Werner and P. J. Knowles, *J. Chem. Phys.*, Vol. 82, Nr. 11, S. 5053–5063, 1985.

[30] P. J. Knowles and H.-J. Werner, *Chem. Phys. Lett.*, Vol. 115, Nr. 3, S. 259–267, 1985.

[31] H.-J. Werner and W. Meyer, *J. Chem. Phys.*, Vol. 73, Nr. 5, S. 2342–2356, 1980.

[32] H.-J. Werner and W. Meyer, *J. Chem. Phys.*, Vol. 74, Nr. 10, S. 5794–5801, 1981.

[33] H.-J. Werner, *Mol. Phys.*, Vol. 89, Nr. 2, S. 645–661, 1996.

[34] P. Celani and H.-J. Werner, *J. Chem. Phys.*, Vol. 112, Nr. 13, S. 5546–5557, 2000.

[35] T. Shiozaki, W. Győrffy, P. Celani, and H.-J. Werner, *J. Chem. Phys.*, Vol. 135, Nr. 8, S. 081106, 2011.

[36] W. Győrffy, T. Shiozaki, G. Knizia, and H.-J. Werner, *J. Chem. Phys.*, Vol. 138, Nr. 10, S. 104104, 2013.

[37] F. Eckert and A. Klamt, *AIChE Journal*, Vol. 48, Nr. 2, S. 369–385, 2002.

[38] A. Klamt, *Wiley Interdiscip. Rev. Comput. Mol. Sci.*, Vol. 1, Nr. 5, S. 699–709, 2011.

[39] F. Liu, N. Luehr, H. J. Kulik, and T. J. Martínez, *J. Chem. Theory Comput.*, Vol. 11, Nr. 7, S. 3131–3144, 2015.

[40] S. Rohani, S. Horne, and K. Murthy, *Org. Process Res. Dev.*, Vol. 9, Nr. 6, S. 858–872, 2005.

[41] B. O. Roos, P. R. Taylor, and P. E. Sigbahn, *Chem. Phys.*, Vol. 48, Nr. 2, S. 157–173, 1980.

[42] M. Head-Gordon, *Chem. Phys. Lett.*, Vol. 372, Nr. 3-4, S. 508–511, 2003.

[43] R. B. Woodward and R. Hoffmann, *J. Am. Chem. Soc.*, Vol. 87, Nr. 2, S. 395–397, 1965.

[44] H. Longuet-Higgins and E. Abrahamson, *J. Am. Chem. Soc.*, Vol. 87, Nr. 9, S. 2045–2046, 1965.

[45] E. F. Hayes and A. K. Siu, *J. Am. Chem. Soc.*, Vol. 93, Nr. 8, S. 2090–2091, 1971.

[46] K. Yamaguchi, *Chem. Phys. Lett.*, Vol. 33, Nr. 2, S. 330–335, 1975.

[47] B. H. Bowser, S. Wang, T. B. Kouznetsova, H. K. Beech, B. D. Olsen, M. Rubinstein, and S. L. Craig, *J. Am. Chem. Soc.*, Vol. 143, Nr. 13, S. 5269–5276, 2021.

[48] M. Ben-Nun and R. Levine, *J. Phys. Chem.*, Vol. 96, Nr. 4, S. 1523–1525, 1992.

[49] S. Izvekov, M. Parrinello, C. J. Burnham, and G. A. Voth, *J. Chem. Phys.*, Vol. 120, Nr. 23, S. 10 896–10 913, 2004.

[50] C. R. Hickenboth, J. S. Moore, S. R. White, N. R. Sottos, J. Baudry, and S. R. Wilson, *Nature*, Vol. 446, Nr. 7134, S. 423–427, 2007.

[51] S. Jung and H. J. Yoon, *Angew. Chem., Int. Ed.*, Vol. 59, Nr. 12, S. 4883–4887, 2020.

[52] I. Coldham and R. Hufton, *Chem. Rev.*, Vol. 105, Nr. 7, S. 2765–2810, 2005.
